# Phylogeographic structure in long‐tailed voles (Rodentia: Arvicolinae) belies the complex Pleistocene history of isolation, divergence, and recolonization of Northwest North America's fauna

**DOI:** 10.1002/ece3.2393

**Published:** 2016-08-29

**Authors:** Yadéeh E. Sawyer, Joseph A. Cook

**Affiliations:** ^1^ Department of Biology and Museum of Southwestern BiologyUniversity of New MexicoMSC03 2020AlbuquerqueNew Mexico87131‐0001

**Keywords:** *Microtus longicaudus*, multilocus, Pacific Northwest, species distribution models, vole

## Abstract

Quaternary climate fluctuations restructured biodiversity across North American high latitudes through repeated episodes of range contraction, population isolation and divergence, and subsequent expansion. Identifying how species responded to changing environmental conditions not only allows us to explore the mode and tempo of evolution in northern taxa, but also provides a basis for forecasting future biotic response across the highly variable topography of western North America. Using a multilocus approach under a Bayesian coalescent framework, we investigated the phylogeography of a wide‐ranging mammal, the long‐tailed vole, *Microtus longicaudus*. We focused on populations along the North Pacific Coast to refine our understanding of diversification by exploring the potentially compounding roles of multiple glacial refugia and more recent fragmentation of an extensive coastal archipelago. Through a combination of genetic data and species distribution models (SDMs), we found that historical climate variability influenced contemporary genetic structure, with multiple isolated locations of persistence (refugia) producing multiple divergent lineages (Beringian or northern, southeast Alaska or coastal, and southern or continental) during glacial advances. These vole lineages all occur along the North Pacific Coast where the confluence of numerous independent lineages in other species has produced overlapping zones of secondary contact, collectively a suture zone. Finally, we detected high levels of neoendemism due to complex island geography that developed in the last 10,000 years with the rising sea levels of the Holocene.

## Introduction

Pleistocene glacial–interglacial cycles repeatedly affected divergence and speciation processes at high latitudes in both the Northern (Koch et al. 2006; Lee‐Yaw et al. 2007; Godbout et al. 2008) and Southern Hemispheres (Lessa et al. 2010). In North America, the Laurentide and Cordilleran ice sheets extended far south during glacial phases (Dyke and Prest 1987; Roberts 1991; Carrara et al. 2007), resulting in major distributional changes in temperate and arctic species (Lyons 2003). As glaciers receded, deglaciated areas were recolonized by populations from refugia (Pielou 1991) and contemporary DNA footprints allow us to decipher the complex history of species movements following deglaciation (Hayes and Harrison 1992; Hewitt 2004; Marr et al. 2012). Populations that colonized de‐glaciated regions have signatures of rapid population expansion (Hundertmark et al. 2002; Lessa et al. 2003; Walker et al. 2009), while refugial populations often reflect long‐term stability (Rand 1954; Shafer et al. 2010; Stewart et al. 2010). Reconstructing recolonization patterns, however, can be problematic (Godbout et al. 2008), especially in topographically complex regions or when sampling is limited, because of the potential for cryptic (unsampled) refugia (Shafer et al. 2010), distinctive modes of expansion (e.g., phalanx and pioneer‐type expansion, Hewitt 1996, 2000), and even selective sweeps when inference is based on a single gene (Excoffier et al. 2009). Furthermore, genetic variation in some recolonized populations may be higher than expected due to admixture between multiple lineages following expansion from independent refugia (Petit et al. 2003; Marr et al. 2008). Admixture may be particularly complex in high‐latitude archipelagos when colonizing populations arise from multiple island source populations (Crawford et al. 2001).

Across the broad expanse of northern North America, a few regions have emerged as sites of high lineage diversity because they are at the confluence of multiple recolonizing lineages (Cook and MacDonald 2013). We focus on one of these regions, the panhandle of southeast Alaska along the North Pacific Coast, where high levels of lineage diversity is being documented for a growing number of species, (e.g., Dawson et al. 2014). These divergent lineages are postulated to have persisted in different ice‐free regions [Beringian, southern continental, or coastal (Pielou 1991; Loehr et al. 2006; Carrara et al. 2007)] with subsequent expansion into proximity (Conroy and Cook 2000; Demboski and Cook 2001). Southeast Alaska, a region with an extensive island archipelago and high levels of endemism (Cook and MacDonald 2001), has been colonized by multiple newly arrived species (Cook and MacDonald 2013). This region also experienced substantial habitat modification in the past 80 years due to commercial logging and other extractive industries (List 2000; Schoen and Dovichin 2007; Albert and Schoen 2013).

We explore how complex topography and dynamic geologic history conspired to shape contemporary lineage diversity in *Microtus longicaudus* (long‐tailed vole), a species that spans more than 35 degrees latitude across western North America in montane and mesic herbaceous habitats (Smolen and Keller 1987; Lomolino et al. 1989). Previous work based on a single mitochondrial gene identified a series of geographically discrete lineages and a single area of secondary contact in southeast Alaska (Conroy and Cook 2000). Divergence among coastal (island) and continental clades was attributed to late Quaternary glacial–interglacial cycling by Conroy and Cook (2000) who suggested the island clade experienced recent expansion from a southeast Alaska refugium with subsequent low gene flow among islands. The northern clade reflected pre‐last glacial maximum (LGM) isolation, while the central and southern clades were the result of deeper (mid‐Pleistocene) divergence.

By employing a multilocus approach and exploring historical climatic conditions predicted by species distribution models (SDM), we test previous phylogenetic hypotheses of lineage diversification across the broad range of *M. longicaudus* (e.g., Conroy and Cook 2000; Spaeth et al. 2009). We then revisit estimates of timing of divergence, refugial locations, and post‐glacial colonization pathways through expanded geographic sampling. By focusing more intensively on the northwestern extent of the species’ range, we first delimit the distribution and source of all lineages in the region. Second, through finer scale sampling, we begin to refine our understanding of the distributional edges of lineages to reveal areas of potential contact between distinct lineages and then test the hypothesis that these sites reflect postglacial (last 10,000 years) contact. Finally, through a combination of genetic data and species distribution models (SDMs), we examine the interplay between historical climate variability and contemporary fragmentation across an island archipelago to set the stage for detailed analyses of diversification and community assembly in this dynamic region.

## Materials and Methods

### Sampling and laboratory procedures

**Figure 1:**
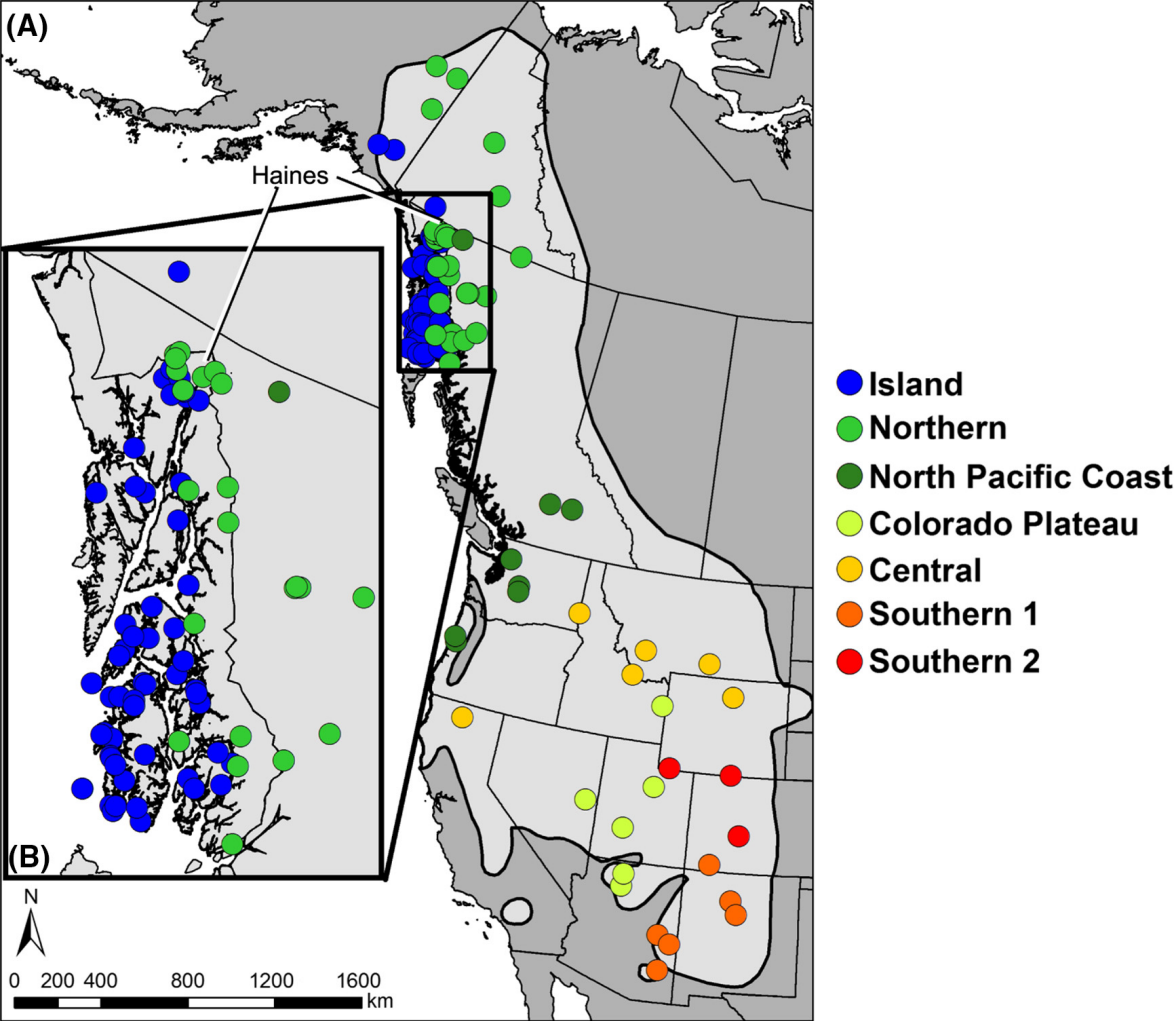
Current range of *M. longicaudus*. Light gray is the current range, black outlined regions. Dots are sampling locations colored by major cyt *b* clades (Fig. S1). (A) Entire range, (B) Haines and southeast Alaska.

Museum specimens were obtained via fieldwork conducted annually since 1991. Frozen heart or liver tissues are archived at the Museum of Southwestern Biology (*n* = 52) and the University of Alaska Museum of the North (*n* = 91). Sampling now represents 46 localities spanning the geographic range of *M. longicaudus*. We focused most intensively on northwestern sampling to explore population structure across the Alexander Archipelago (AA) of southeast Alaska and identify the extent of contact between island and northern clades in southeast Alaska, with a higher sampling density near Haines (*n* = 28; Appendix S1; Fig. 1). Samples represented 11 of the 15 currently recognized subspecies of *M. longicaudus* (Fig. 1; Hall 1981). Additional cytochrome *b* gene (cyt *b*) sequences were obtained from GenBank for 67 individuals of *M. longicaudus*, one for each outgroup (*M. pennsylvanicus* and *M. montanus*), as well as a single Rag1 sequence for *M. pennsylvanicus* (Appendix S1).

Genomic DNA was extracted using Omega Bio‐Tek (Norcross, GA) E.Z.N.A. kits or salt extraction (Fleming and Cook 2002), with final concentrations adjusted to 50 ng *μ*L^−1^. We amplified mitochondrial (mtDNA) cyt *b* (767–1143 bp, *n* = 118) and nuclear (nuDNA) gene sequences, including Protein C‐est‐2 (ETS2, 731 bp, *n* = 71), *β*‐fibrinogen (FGB, 600 bp, *n* = 101), and recombination‐activating protein 1 (Rag1, 1059 bp, *n* = 83; Table S1). Polymerase chain reaction, sequencing, visualization, editing, and phasing procedures for alleles were detailed in Sawyer (2014). All sequences were deposited in GenBank (Appendix S1).

### Phylogenetic analyses and estimation of divergence times

We used a multilocus approach to reconstruct species relationships (Maddison 1997; Carstens and Knowles 2007; Edwards et al. 2007). The species tree was estimated in *beast (Heled and Drummond 2010), which uses a Bayesian Markov chain Monte Carlo (MCMC) coalescent approach to coestimate multiple gene trees embedded within the corresponding species tree topology. *A priori* groups were based on geographic populations, with individuals within northern populations assigned based on cyt *b*‐supported clades (Appendix S1 – “locality”). Independent, unlinked loci (mtDNA and each nuDNA locus) were partitioned by gene and set to appropriate substitution models (Table S2) calculated in modeltest. An *a priori* uncorrelated lognormal relaxed clock was set for cyt *b* with a mutation rate of 4% Myr^−1^ based on previous estimates ranging from 3 to 5% Myr^−1^ (Conroy and Cook 1999; Brunhoff et al. 2003; Hope et al. 2013). Utilizing a Bayesian uncorrelated relaxed clock assists in reducing errors associated with recent divergence and the lack of reliable calibration points (Drummond et al. 2006; Ho and Duchene 2014). The tree priors were set to a Species Tree Yule Process with a piecewise linear and constant root population size model and random start tree. MCMC chain was run for 2 billion iterations, sampling every 2 million. Time to most recent common ancestor (TMRCA) was determined with a 95% posterior probability distribution in tracer v1.5 (Rambaut & Drummond 2007). For each tree, convergence statistics were assessed with effective sample size (ESS) values ≥200 in tracer and convergence onto the same tree was analyzed with the online software awty (Nylander et al. 2008). Three independent runs were checked for convergence in trace graphs then combined using logcombiner v1.7.5, with a 10% burn‐in. Tree files were annotated in treeannotator v1.7.5, and topologies were visualized in figtree v1.4.0 (Rambaut 2009). Because we lacked reliable fossils for this species, we were unable to incorporate fossil calibration into either cyt *b* (Data S1) or species tree analyses.

### Migration estimates

We used bayesass v3.0.3 (Wilson and Rannala 2003) to determine recent levels of gene flow among populations representing the major cyt *b* clades (designated with a 95% support for Bayesian and 70 for ML; Fig. S1) across the islands of southeast Alaska, as well as among northern populations with secondary contact or in close geographic proximity. bayesass uses a nonequilibrium, multilocus Bayesian approach to estimate migration rates under a MCMC algorithm. We used phased multilocus data (mtDNA and nuDNA) and ran 200 million iterations with a 20 thousand burn‐in and sampling every 2 thousand. Mixing parameters of allele frequencies, inbreeding coefficient, and migration rates were adjusted following program guidelines.

### Demographic analyses

To explore the signatures of stability and postglacial expansion for each well‐supported major cyt *b* clade, we reconstructed extended Bayesian skyline plots (EBSP; mtDNA and nuDNA) and cyt *b* Bayesian skyline plots (Heled and Drummond 2008) implemented in beast. Colorado Plateau (COP) and North Pacific Coast (NPC) were omitted due to low sample sizes. Strict molecular clocks for all phased loci and appropriate models of evolution (Table S2) assigned for each of three independent runs per data set included a MCMC chain of 2 billion steps, sampled every 2 million steps. tracer was used to assess convergence. Significant population size change occurred in EBSPs if zero was excluded from the 95% confidence interval (CI) of the estimate of the number of size‐change steps (Lim and Sheldon 2011). To test for recent demographic fluctuation in cyt *b* major clades for each locus, we calculated a series of population genetic summary statistics [segregating sites (*S*), haplotype diversity (*Hd*), and nucleotide diversity (*π*)] for both mtDNA and nuDNA in dnaSP 5.10.1 (Librado and Rozas 2009). Historical demographic change or selection potential were assessed through Tajima's *D* (1989), Fu's *Fs* (1997), and Ramos‐Onsin and Rosas’ *R*_*2*_ (Ramos‐Onsins and Rozas 2002) with 10 thousand coalescent simulations. Selection potential was assessed through the HKA test (Hudson et al. 1987).

### Species distribution models

We generated SDMs for *M. longicaudus* to identify regions of climate suitability across western North America, which were then tested with molecular data. Because we used relatively few southern samples, we did not generate clade‐specific models. Models included bioclimatic variables obtained from worldclim (www.worldclim.org, Hijmans et al. 2005) at a resolution of 2.5 arc‐minutes for current, as well as mid‐Holocene (~6 ka) and last glacial maximum (LGM; ~21 ka, http://pmip2.lsce.ipsl.fr/, Braconnot et al. 2007), and the last interglacial (LIG; ~120–140 ka). To avoid over‐parameterization of the model, we used ENMtools (Warren et al. 2008, 2010) to eliminate highly correlated variables (Pearson correlation ≥0.75). Test layers were clipped to the extent of sampling, while projection layers were for western North America. Correlated bioclimatic variables were removed and only those judged to be the most important to *M. longicaudus* were retained for the final runs. Locality data were obtained from museum databases (e.g., ARCTOS http://arctos.data-base.uaf.edu and MaNIS http://manisnet.org/; Stein and Wieczorek 2004). Sites with large (>50 km) spatial errors were removed. Although genetic sampling was denser in the northern latitudes, that was not the case for SDMs (Fig. S2). To moderate spatial autocorrelation (Reddy and Davalos 2003), we reduced sample points to 50 km apart by removing intervening samples (Hope et al. 2011) for 149 final localities.

Species distribution models were constructed for each time period using maxent v3.3.3k (Elith et al. 2006; Phillips et al. 2006; Phillips and Dudik 2008). Basic assumptions were as follows: no topographic change has occurred, niche conservatism (Wiens and Graham 2005), environmental data adequately predict species occurrence (Kozak et al. 2008; McCormack et al. 2010), and sampling records effectively captured the entire niche breadth of the species (Pearson et al. 2007). Final runs used bioclimatic variables 1 (annual mean temperature), 6 (min temperature of coldest month), 7 (temperature annual range), 9 (mean temperature of driest quarter), and 11 (mean temperature of coldest quarter) and were performed using cross‐validation across 10 runs, with a random test percentage of 25%, regularization parameter of 5 (e.g., Hope et al. 2011; Warren and Seifert 2011), and 1000 iterations; all other values were default. Models were tested for performance using the randomization feature in ENMtools. Mean and median models were not significantly different from each other, so mean models based on MIROC and CCM models of LGM were averaged in arcGIS 10.1 (ESRI, Redlands, CA) using the raster calculator. Minimum threshold values for climate suitability were the low median threshold values over all replicates (Pearson et al. 2007). These models were assessed with respect to potential refugial locations as identified by Sawyer (2014).

## Results

### Phylogenetic analysis

**Figure 2:**
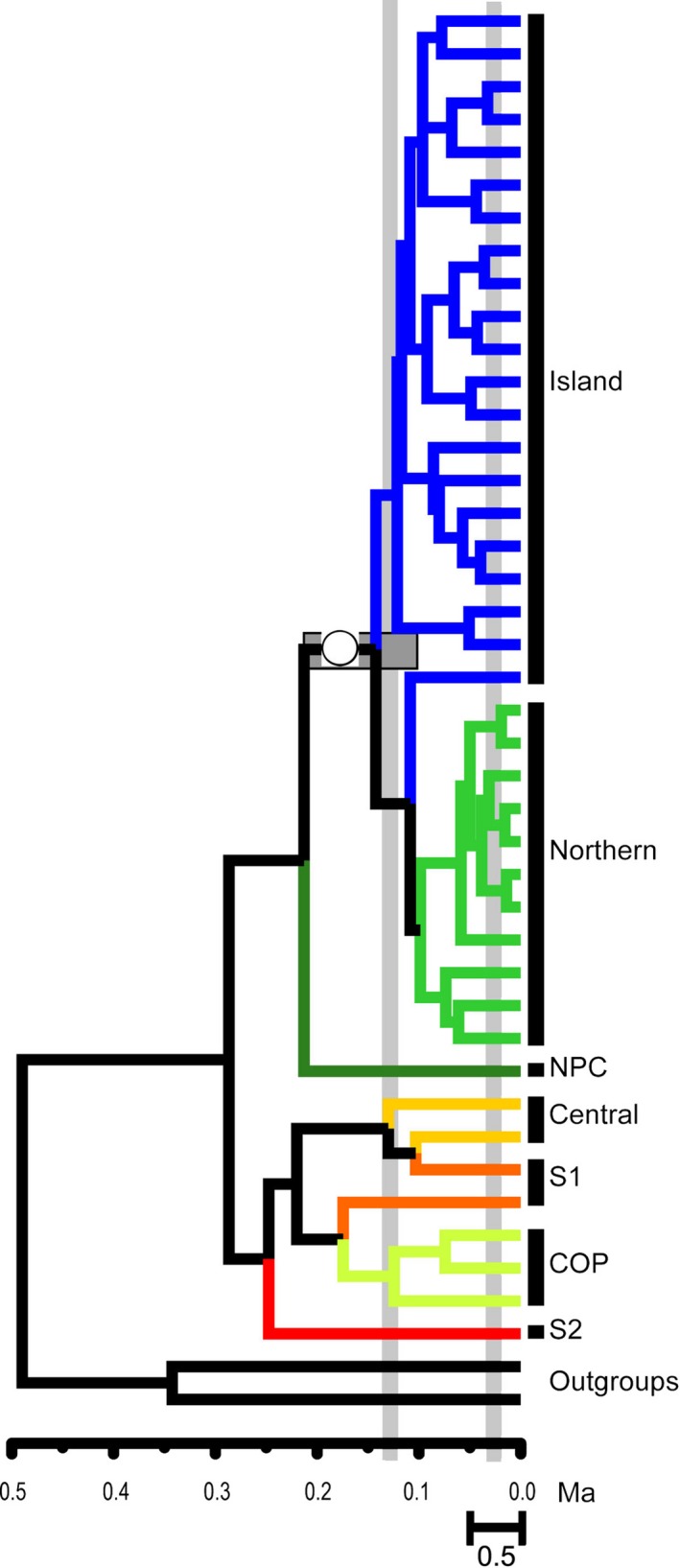
Phased multilocus Bayesian Species Tree. Posterior probabilities of ≥0.95 are represented with open circles on branches. Horizontal gray bars represent divergence date estimates and vertical bars indicate approximate time for the LIG and LGM. NPC = North Pacific Coast; COP = Colorado Plateau; S1 & S2 = southern clades.

The multilocus species tree identified just a single well‐supported clade (island and northern together; Fig. 2), while other clades identified based on mtDNA alone (Conroy and Cook, 2000; this study) were only moderately supported. Nuclear haplotypes (Fig. S3) generally were either lineage specific or shared across many individuals. Shared alleles were not dependent on geographic proximity, suggesting the lower resolution found in the species tree compared to the more geographically structured mtDNA tree may result from incomplete lineage sorting, rather than admixture (Toews and Brelsford 2012).

We found stronger geographic structure within the faster evolving cyt *b* across the range of *M. longicaudus* (Fig. 1 and Fig. S1)*,* but all phylogenies were consistent with four geographic groups: (1) southern, comprised of two lineages (S1: Colorado and Wyoming; S2: New Mexico, Colorado, and Arizona); (2) central (California, Idaho, Montana, and Wyoming); (3) COP (Arizona into Idaho); and 4) northwest, consisting of NPC (British Columbia, Oregon, and Washington), northern (eastcentral Alaska, through Yukon and British Columbia), and island (southcentral Alaska, southern Yukon, and southeast Alaska). Two mainland southeast Alaska locations, Haines and Juneau, have representatives of both northern and island clades.

Both northern and island clades showed substantial internal structure, with many geographically restricted lineages identified (Fig. 2and Fig. S1).

### Genetic diversity, demographic analyses and current levels of gene flow

**Figure 3:**
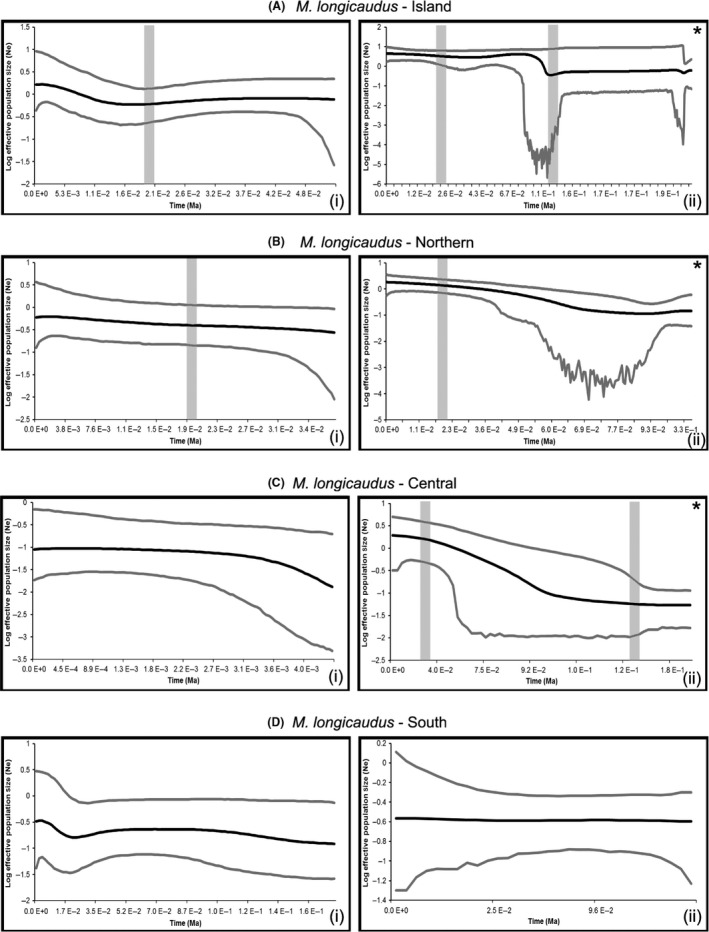
Cyt *b* Bayesian skyline plots (i) and EBSPs (ii) for the major cyt *b* clade populations: (A) island, (B) northern, (C) central, and (D) southern, excluding NPC and COP. EBSP central line indicates mean change in effective population size through time, with upper and lower lines showing the 95% posterior density. Significant population size change in EBSPs are indicated with asterisks. The x‐axis right‐to‐left from past (TMRCA) to present and is scaled in millions of years and the y‐axis is the log effective population size scaled by generation time. Vertical gray bars indicate the LIG (when applicable, right) and LGM (left) for reference.

Nuclear loci had varying levels of allelic diversity (Table S2), with ETS2 the most geographically structured. No selection was detected in HKA tests for all loci. Ramos‐Onsin and Rozas *R*_*2*_ values were significant for all loci. We inferred demographic history based on degree of genetic variation, significance of expansion statistics (Table S2), and both cyt *b* skyline plots and EBSPs (Fig. 3). Populations that experienced recent expansion generally have low *Hd,* while high *Hd* and *π* are indicative of long‐term stability, and low *Hd* and high *π* suggest population bottlenecks. Levels of variation across all loci (mtDNA and nuDNA), although generated independently, were assessed for consistency across loci. The genetic signature of the island clade is consistent with a founder event that then experienced rapid growth, with high cyt *b Hd* a result of genetic drift in the small, fragmented populations of the archipelago. The northern clade signature is also consistent with reduced ancestral population size followed by rapid expansion, while the central clade was historically stable. The southern clade represents a glacially stable population that may have experienced a bottleneck (this signature only in the cyt *b* data). Small sample sizes for the NPC and COP clades preclude inference.

The mean of three runs for the Bayesian estimates of migration indicated low levels of gene flow between major cyt *b* clades within northern regions of secondary contact (Table 1), with the island clade the most genetically isolated. Gene flow (proportion of migrants derived from other populations) among geographically proximate representatives of distinct clades was highest from populations in the northern clade (Haines and Juneau) into the Haines population of the island clade (0.009–0.012; Fig. S4; Table S5). The island clade contained populations with a very small proportion of mainland migrants (<0.0187 between any island/adjacent mainland pair; Table S6).

**Table 1:** Major cyt*b* clade in regions of northern secondary contact. Bayesian migration estimates determined in bayesass. Nonmigrants within each population are indicated in bold along the diagonal. Values are the proportion of migrant genes donated from source populations (columns) into sink populations (rows)

From…	Migration rates into…		
	Island	Northern	NPC
Island	**0.988**	0.007	0.005
Northern	0.019	**0.970**	0.011
NPC	0.050	0.156	**0.795**

### Divergence times and alternate models of glacial refugia and postglacial recolonization

The multilocus estimation places the north/island clade TMRCA before the LIG (142 ka; Table S7; Cyt *b* estimates – Data S1).

**Figure 4:**
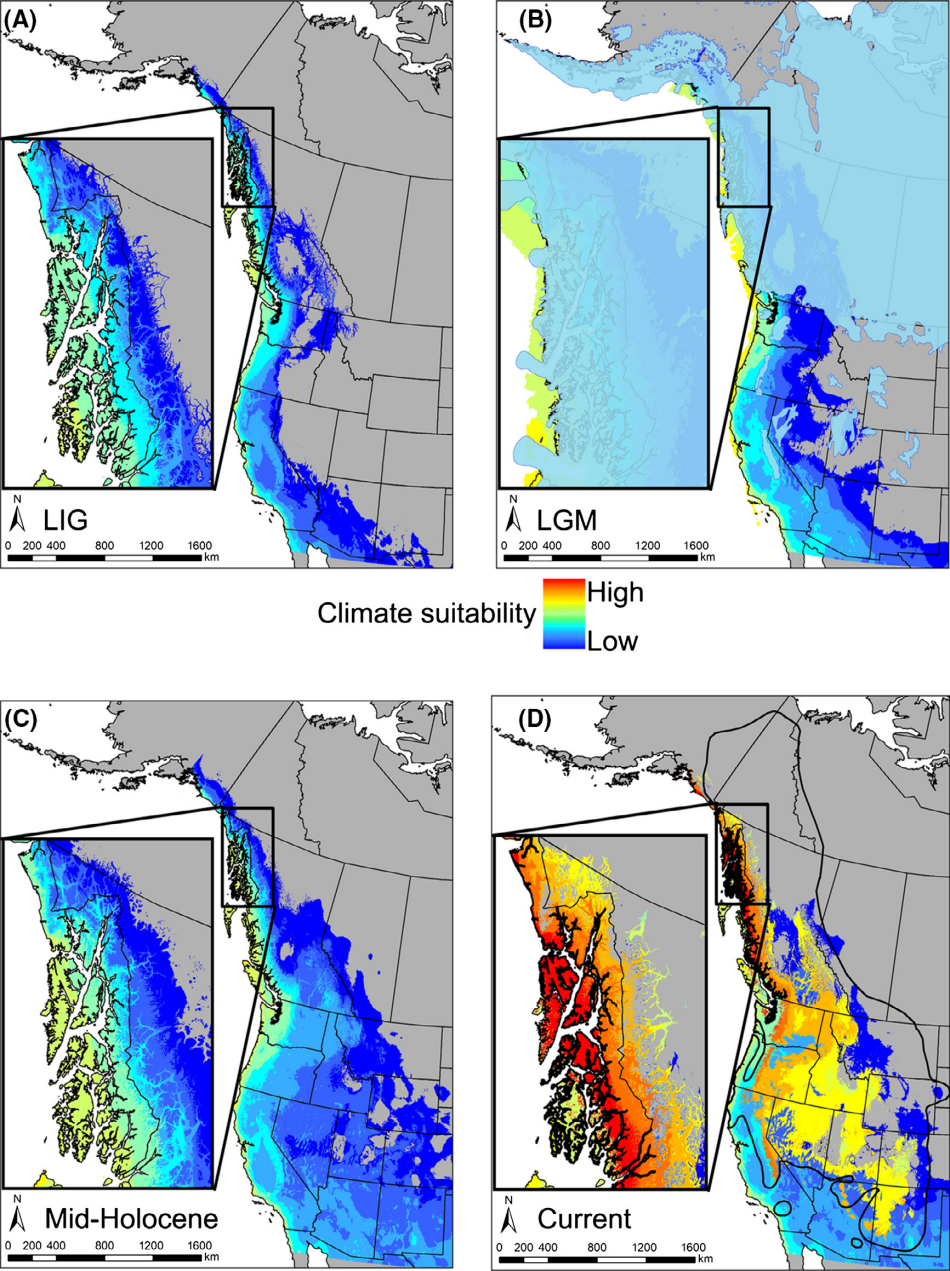
SDM output. (A) LIG (~125 ka), (B) LGM (~20 ka), (C) mid‐Holocene (~6 ka), and (D) current time periods. The thick black line in (D) is the current range for *M. longicaudus*. The solid blue coloring in (B) is glacial ice cover. SDM climate suitability at each time period is limited by minimum median threshold values over all replicates. Because the SDMs are conservative, not all current localities are predicted in the models.

Strong predictive ability of SDMs is indicated with AUC values >0.80 (Warren et al. 2008). SDMs in this study had predictive performances with a mean AUC value of 0.834 (standard deviation 0.04) for the training and test data and performed significantly better than random. The LIG SDM suggests *M. longicaudus* was restricted to coastal or extreme southern locations at this time, with a subsequent increase in climate suitability for the LGM in interior western North America, allowing northward and eastward movement. By the mid‐Holocene, climate conditions were suitable to cover most of the current range of *M. longicaudus*. Because SDMs are conservative (i.e., limited by a minimum threshold value) and relatively few northern continental localities were included, the full northern range currently occupied was not predicted in the models (Fig. 4).

## Discussion

Deciphering the dynamic history of a region sets the stage for interpreting how contemporary environmental change (e.g., deforestation, climate change) may impact species and ultimately ecosystems. In the case of coastal Alaska, our understanding of diversity, island biogeography, and community assembly across the vast Alexander Archipelago was predicated on the hypothesis that the region was entirely glaciated until about 12,000 years ago, and this suggested a narrow timeframe for colonization and diversification of the contemporary biota (Klein 1964). Subsequent phylogeographic (Cook et al. 2006; Shafer et al. 2010), geological (Carrara et al. 2007; Ager et al. 2010), and paleontological (Heaton and Grady 2003, 2007) investigations have revealed complex histories including deeper, in situ persistence and divergence for many species along the coast that were later followed by waves of colonization westward through the coast range from other ice‐age refugia (Cook and MacDonald 2013).

Through the use of multilocus phylogenetics and SDMs, we explored how historical climatic events structured genetic variation in a common rodent across northwestern North America and then focused more intently on subsequent diversification across the islands of southeast Alaska. Sources for postglacial colonization of these northern latitudes typically were located in either southern ice‐free regions (northward; e.g., Stone et al. 2002) or Beringia (southward; e.g., Eddingsaas et al. 2004). Because populations of taxa in the higher latitude deglaciated regions are hypothesized to be relatively homogenous due to recent northward expansion while southern source populations should retain signatures of long‐term persistence and deeper divergence (Hewitt 2000; Lessa et al. 2003; Malaney and Cook 2013), we expected to find similar genetic signatures for *M. longicaudus*. Instead, we uncovered substantial structure in coastal Alaska, with admixture in some mainland populations that reflected complex histories of origin. Contemporary coastal mainland populations are derived from historically persistent (coastal refugial) populations in Southeast Alaska (Carrara et al. 2007) or derived from recent westward colonizers that arrived from one or more refugial sources outside of the region. Contemporary island populations in contrast are derived only from populations that persisted in or near the region (i.e., coastal refugium) and each of these insular populations subsequently has begun to diverge independently (neoendemics). We emphasize persistence “in or near the region” because the precise location of a LGM coastal refugium or series of refugia, when sea levels were up to 165 m lower, has not been identified. Intraspecific variability in this species at the far northwestern edge of its range mirrors to some extent the history of several other species studied to date, indicating that contemporary species in the region may be admixed as a result of contact between distinctive persistent and colonizing lineages. Similarly, contemporary communities in the region are a composite of both persistent and recently colonized species that arrived from multiple geographic sources (Cook et al. 2001, 2006). This dynamic perspective on assembly where species and communities may have multiple origins has broad implications ranging from new perspectives on the island biogeography of the archipelago, to revising conservation plans for this highly fragmented and heavily altered landscape and to establishing the history and critical roles of parasites in wildlife and zoonotic pathogens in humans in the region.

### Biogeographic drivers of isolation, glacial refugia, and postglacial recolonization

**Figure 5:**
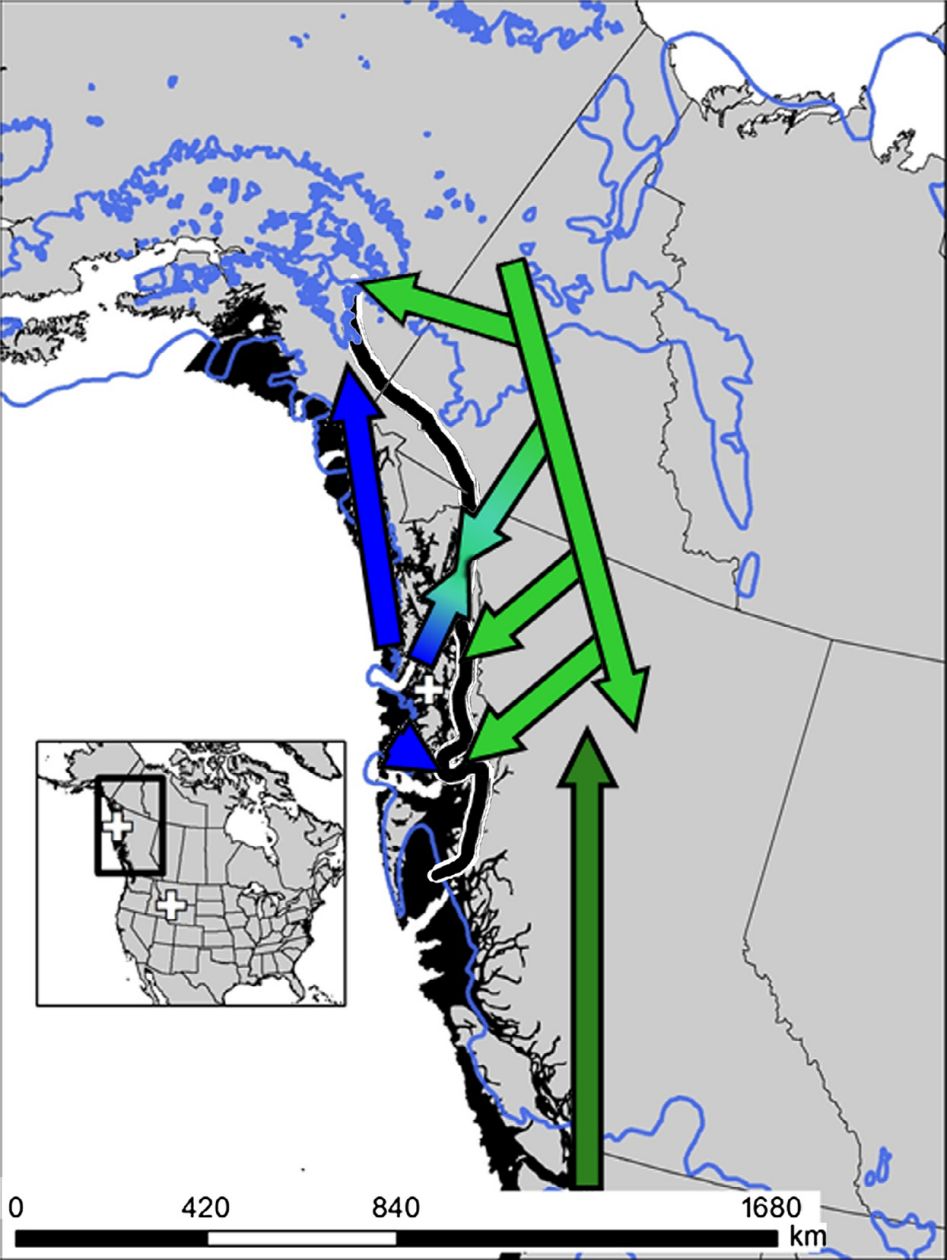
Proposed postglacial colonization routes (arrows) for *M. longicaudus* based on genetic signatures and SDMs. Plus (+) = fossil (SE Alaska)/subfossil (Yellowstone) locations and arrow colors correspond to current cyt *b* lineages: dark green = North Pacific Coast, green = northern, and blue = island. Northern and island clade refugial locations and postglacial colonization with location of geographic proximity indicated with the thick black line and secondary contact with converging arrows. Blue line indicates extent of glacial ice, and solid black coastal regions are exposed continental shelf at the LGM.

This more detailed view of the phylogeography of *M. longicaudus* refines our understanding of divergence within southeast Alaska and largely corroborates earlier continental‐scale work that revealed patterns of differentiation based on more restricted geographic and genetic sampling (Conroy and Cook 2000; Spaeth et al. 2009), but there are notable discrepancies. Spaeth et al. (2009) focused on the Greater Yellowstone Ecosystem, identifying postglacial subfossils representing both the northern and central clades within Yellowstone (Fig. 5). Our expanded assessment detected substantial spatial and temporal patterns of genetic differentiation across the entire range of *M. longicaudus,* not previously identified by either Conroy and Cook (2000) or Spaeth et al. (2009).

The multilocus estimate for TMRCA of *M. longicaudus* is before the LIG (about 290 ka, Fig. 2), but is shallower than single‐locus estimates from previous studies (340 ± 70 ka, Conroy and Cook 2000). Difficulty in establishing these estimates may reflect our inability to adequately calibrate trees with fossils and thus account for rate decay (Ho et al. 2005). Contrasting with the pre‐LIG mtDNA estimates of divergence (Fig. S3), the shallower fossil dates are extending only to the Wisconsinan glaciation in Idaho, Wyoming, Colorado, and New Mexico (Schultz and Howard 1935; Anderson 1968; Harris 1970; Hager 1972; Walkerd 1982; White et al. 1984) and postglacial locations in Alberta and British Columbia (Harington 2011b). The absence of older fossils, especially at the northern extent of their range where we suspect refugial persistence (Guthrie 1968; Youngman 1975), may reflect poor preservation, lack of exploration of the region, or simply the difficulty of distinguishing vole fossil teeth (Harington 2011a). If the earlier DNA‐derived estimate for initiation of separation (290 ka) is correct, then divergence among major lineages of *M. longicaudus* was likely reinforced through late‐Pleistocene cycles of glacial isolation, as indicated by the SDMs (Fig. 4). Southern clades apparently experienced longer‐term stability and isolation, while the strongly supported, yet shallow structure among most northern clades is due to recent (Holocene) range expansion.

Our findings are consistent with the hypothesis that long‐tailed voles persisted in a southeast Alaska coastal refugium (island clade) during the LGM, followed by expansion onto the islands and mainland and then recent isolation on multiple islands as sea levels rose (Fig. 5). Heaton and Grady (2007) found abundant *M. longicaudus* fossils from On Your Knees Cave (49‐PET‐408) on Prince of Wales Island (Fig. 5) that radiocarbon date to the middle Wisconsin Interstadial (38–25 ka), but fossil evidence disappeared by the LGM due to glacial advance onto this island. *Microtus longicaudus* reappeared in On Your Knees and El Capitan and Bumper caves on Prince of Wales Island by the early Holocene (Heaton and Grady 2003), which suggests rapid recolonization shortly after the LGM. The number of coastal refugial sites occupied by *M. longicaudus* should be further explored because SDMs and multilocus genetic signatures suggest the possibility that *M. longicaudus* persisted in up to three refugia within the AA. Fossil support for refugial locations in the AA may prove problematic, however, as much of the coast exposed during the LGM is now under up to 165 m of water (Carlson 2007; Shugar et al. 2014).

Lack of connectivity between insular populations produced extensive interisland structure, with populations on 13 of the 19 islands displaying island‐specific haplotypes and significant mtDNA divergence (see also Sawyer 2014). Gene flow among island populations in the AA was inhibited due to sea‐level rise during the early Holocene (c. 14–8 ka bathymetric reconstruction, Baichtal and Carlson 2010). Also, within the AA, *M. longicaudus* is the only vole that occurs on most of the islands (Baranof island is a curious exception). On the mainland of southeast Alaska, this vole is syntopic with two congeners, *M. oeconomus* (root vole) and *M. pennsylvanicus* (meadow vole); however, the phylogeographic histories of those two species remain poorly understood in the region. Both species are thought to be Holocene colonizers of southeast that originated outside of the coastal region (Cook and MacDonald 2013). With regard to other potentially closely associated species, the phylogeography of the ermine (*Mustela erminea)*, a vole specialist predator (Verts and Carraway 1998), has been explored. Three distinctive lineages of ermine, one endemic (island) and two originating outside the region (Beringian and southern) also have been identified in southeast Alaska (ermine; Fleming and Cook 2002; MacDonald and Cook 2007; Dawson et al. 2014), although there is limited spatial correspondence between the long‐tailed vole and ermine lineages at finer spatial scales within southeast Alaska. The island lineage of *M. longicaudus,* for example, co‐occurs with all three major clades (continental, Beringian, and island) of ermine. Hence, although the broader regional patterns of lineage divergence due to persistence in multiple glacial refugia are shared among this predator and its prey, idiosyncratic differences due to distinct colonization abilities, lineage persistence, or random events are evident on individual islands and on the mainland in southeast Alaska. These limited examples point to the need for more detailed comparative assessments with other taxa, such as plants and associated arthropods, across this complex landscape.

The island and northern clades of *M. longicaudus* have at least two points of secondary contact on the mainland (Haines and Juneau) and the multiple sites of geographic proximity southward along the mainland coast of southeast Alaska indicate the possibility of additional locations of contact between distinctive lineages (Fig. 4). Island individuals likely followed glacial retreat through multiple colonization pathways (Fig. 5) to recolonize the adjacent mainland while sea levels were low, while northern individuals apparently colonized the mainland rapidly through river corridors extending through the coast range after ice sheets receded. Detailed assessment of mainland sites based on increased sampling density is needed to interpret the expansion from the east, a process noted for other mammals (Cook and MacDonald 2013). This region of contact along the coast may reflect a generalized suture zone, where processes of introgression are similar to those detected in coastal populations of red‐backed voles of the genus *Myodes* (Runck et al. 2009) and montane shrews, *Sorex monticolus* (Demboski and Cook 2001; Sawyer 2014). Contrary to previous suggestions that the northern clade of long‐tailed voles expanded along a single interior route from south of the ice (Conroy and Cook 2000), we hypothesize that the northern clade persisted in Beringia and recently expanded southward into previously glaciated regions of southern Alaska, Yukon, and northern British Columbia (Fig. 5) as noted in moose, wolverine, ermine, and other species (MacDonald and Cook 2007). Two points that are potentially inconsistent with this conclusion are (1) lack of verifiable pre‐Holocene fossils of long‐tailed voles in Beringia, and (2) SDMs do not predict suitable environments north of the ice for long‐tailed vole persistence during the LGM. We also recognize the possibility that with increased sampling across Yukon and British Columbia, either a more southern distribution of the northern clade may be detected, increasing the possibility of a southern (vs. Beringian) origin for this clade, or climatic niche suitability with a more northern range may be detected, supporting the Beringian origin for this clade. High levels of intralineage genetic diversity, net genetic distance among other clades, and the restriction of the range of this clade to high latitudes, however, are consistent with persistence in a northern refugium. Additionally, the species tree indicates a close association of the island and northern clades, suggesting a northern origin. Numerous paleontological as well as phylogeographic studies that identified genetic signatures point toward the persistence of many species in an eastern Beringia refugium during glacial periods, but clearly, there is a need for expanded analyses to further refine our understanding of these northern communities during the LGM (Fleming and Cook 2002; Stamford and Taylor 2004; Sawyer 2014).

Species distribution models are consistent with persistence of *M. longicaudus* along the coast of Oregon and Washington (NPC clade) during glacial advances. The central and southern clades were effectively isolated and relatively demographically stable since the mid‐ to late‐Pleistocene. Lastly, the COP clade, previously identified from a limited region in northern Arizona, was found to occur northward into Utah and potentially contacts both the S2 subclade and central clade.

### Contemporary genetic structure and current levels of gene flow

Deciphering signatures of incomplete lineage sorting versus secondary admixture is critical for interpreting how historical climate shaped contemporary distributions and genetic structure. Shallow divergence between northern and island populations near Haines may reflect recent (Holocene) secondary contact or incomplete lineage sorting. Future studies should incorporate more intensive sampling of individuals, especially in suspected contact zones, and expanded genomic coverage to explore potential admixture (Qu et al. 2012).

The distribution of major clades in *M. longicaudus* is generally inconsistent with subspecific designations (Fig. 1; Hall 1981). For example, Forrester and Coronation are identified as independent lineages, but these islands were previously placed in a single subspecies, *M. l. coronarius* (MacDonald and Cook 2009). All major clades span multiple subspecies of *M. longicaudus*, while several subspecies also include multiple clades. A reassessment of subspecies, originally based on morphological variation, is warranted.

Segregation of the northern/island clade from the NPC, COP, central, and southern clades is reflected in both the species tree (Fig. 2) and migration estimates (Table 1). The lack of support for deeper nodes may be a result of the relatively dense sampling for the northern/island clade. Compared to the northern clade, lower levels of gene flow from the island clade into the Haines area likely reflect physical barriers to movement (i.e., ocean straits, extant glaciers). Genetic exchange among island populations also is now limited, a finding that points to the possibility that these insular biotas are evolving independently and further reinforcing the urgency of managing this archipelago on an island by island basis, given that the AA comprises the largest federally managed forest in the United States, the Tongass National Forest (6.8 million ha).

## Conclusions

Understanding the interplay between topography and historical climate change on both the distribution of contemporary species and the structure of genetic variation within these species sets, a powerful stage for forecasting and mitigating biotic responses to changing conditions. At a regional scale along the long‐tailed vole's boundary in the Pacific northwest, significant structure reflects their dynamic expansion from multiple refugia concomitant with the contraction of glacial cover and as influenced by rising sea levels and isostatic rebound that played out over the complex topography of Pacific coastal North America. Similar processes of isolation and complex colonization along the North Pacific Coast were recently identified for this vole's primary predator, the ermine (Dawson et al. 2014). Our expanded view of molecular variation on 19 islands across the AA identifies the deeper signature of persistence in a coastal refugium that was the source for continental admixed populations and insular populations that are subsequently diverging, largely independently. Further work should focus on how species on individual islands will respond to changing environmental conditions, including a series of anthropogenic stressors (Hannon et al. 2010; Cook and MacDonald 2013) and rising sea levels. Recovering ancient DNA from the extensive fossil record from the region (>3000 specimens; T. H. Heaton, pers. comm.) would significantly extend our comprehension of temporal genetic variability (Miller et al. 2012) in this dynamic region.

## Data accessibility

Sample locations with museum numbers, latitude and longitude, major cyt *b* clade, and associated GenBank accession numbers are uploaded as online supplemental material in Appendix S1. PCR conditions are provided as Supporting information in Table S1.

## Conflict of Interest

None declared.

## Supporting information


**Appendix S1.** Specimens examined.

**Figure S1.** Dated Bayesian cyt*b* trees.
**Figure S2.** Sampling localities for species distribution models.
**Figure S3.** Phased Bayesian gene trees for (A) ETS2, (B) FGB, and (C) Rag1 nuclear loci with posterior probabilities of ≥0.95 represented with open circles on branches of the solid consensus tree.
**Figure S4.** Phased nuclear haplotype distribution in Northern and Island clades of *M. longicaudus*.
***Table S1.** Primer list and PCR annealing temperatures.*
***Table S2.** Diversity indices, expansion statistics and models of evolution.*
***Table S3.** Between group net genetic distance.*
***Table S4.** Locality abbreviations.*
**Table S5.** Island and Northern cyt*b* clade populations near the geographic regions of contact (Haines and Juneau, Alaska).
**Table S6.** Bayesian migration estimates for Southeast Alaska populations determined in bayesass for *M. longicaudus*.
**Table S7.** Cyt *b* and phased multilocus divergence date estimates.
**Data S1.** Cyt *b* data ‐ Methods and Results.
