# Supplementary material for: Phylogeographic structure in long‐tailed voles (Rodentia: Arvicolinae) belies the complex Pleistocene history of isolation, divergence, and recolonization of Northwest North America's fauna
Source: Ecol Evol. 2016 Aug 29;6(18):6633–47. doi: 10.1002/ece3.2393 (PMC5058534; doi:10.1002/ece3.2393)
Supplement: Supplementary file 1 — Appendix S1. Specimens examined. [file ECE3-6-6633-s001.docx]

Appendix I. Specimens examined. Major cyt*b* clade C=Central, I=Island, and N=Northern. Museum number acronyms are MSB= Museum of Southwestern Biology; UAM=University of Alaska Museum of the North, Fairbanks. GenBank numbers correspond to cyt*b*, and each allele for ETS2, FGB, and Rag1, respectively, – = not applicable. GenBank sequences in bold were obtained from other studies. Outgroup sequences for FGB and *M. montanus* sequences for ETS2 and Rag 1 were not generated

| **cyt *b* clade** | **Specimen Number** | **Locality** | **Latitude** | **Longitude** | **GenBank Accession Numbers** |
| --- | --- | --- | --- | --- | --- |
| Central (C ) | UAM77991 | California | 41.409 | -122.194 | **AF187176**, – / – , – / – , – / – |
|  | UAM77992 |  | 41.409 | -122.194 | **AF187174**, – / – , – / – , – / – |
|  | UAM77993 |  | 41.409 | -122.194 | **AF187175**, – / – , – / – , – / – |
|  | UAM77994 |  | 41.409 | -122.194 | **AF187177**, – / – , – / – , – / – |
|  | MSB227995 | Idaho | 44.659 | -113.216 | KF948609,KF948677/KF948678,KF948845/KF948846,KF949027/KF949028 |
|  | UAM77997 |  | 46.732 | -117.000 | **AF187173**, – / – , – / – , – / – |
|  | MSB156351 | Montana | 45.731 | -112.676 | KF948534, – / – , – / – , – / – |
|  | UAM34299 |  | 45.520 | -108.820 | **AF187172**, – / – , – / – , – / – |
|  | MSB225915 | Wyoming | 44.226 | -107.235 | KF948607,KF948673/KF948674,KF948841/KF948842,KF949023/KF949024 |
| Colorado Plateau (COP) | MSB61434 | Arizona | 35.954 | -112.157 | **AF187162**, – / – ,KF948853/KF948854, – / – |
|  | MSB61436 |  | 35.954 | -112.157 | **AF187161**, – / – ,KF948855/KF948856, – / – |
|  | MSB61443 |  | 36.463 | -112.118 | **AF187160**, – / – ,KF948857/KF948858,KF949033/KF949034 |
|  | MSB143745 | Idaho | 43.553 | -111.243 | KF948533, – / – ,KF948785/KF948786, – / – |
|  | MSB227146 | Nevada | 39.238 | -114.689 | KF948608,KF948675/KF948676,KF948843/KF948844,KF949025/KF949026 |
|  | MSB76827 | Utah | 38.344 | -112.491 | KF948610, – / – ,KF948859/KF948860, – / – |
|  | MSB77127 |  | 40.186 | -111.140 | KF948611,KF948683/KF948684,KF948861/KF948862,KF949035/KF949036 |
| Island (I) | UAM30499 | Alaska - Glacier Bay | 58.450 | -135.917 | **AF187225**, – / – ,KF948897/KF948898,KF949071/KF949072 |
|  | MSB193312 | Alaska - Haines | 59.218 | -135.448 | KF948589, – / – ,KF948807/KF948808, – / – |
|  | MSB193325 |  | 59.267 | -135.603 | KF948590, – / – ,KF948809/KF948810,KF948997/KF948998 |
|  | MSB193328 |  | 59.267 | -135.603 | KF948591,KF948655/KF948656,KF948811/KF948812,KF948999/KF949000 |
|  | MSB193329 |  | 59.267 | -135.603 | KF948592,KF948657/KF948658,KF948813/KF948814,KF949001/KF949002 |
|  | MSB193396 |  | 59.218 | -135.448 | KF948593,KF948659/KF948660,KF948815/KF948816,KF949003/KF949004 |
|  | MSB193439 |  | 59.267 | -135.603 | KF948594,KF948661/KF948662,KF948817/KF948818,KF949005/KF949006 |
|  | MSB193445 |  | 59.267 | -135.603 | KF948595,KF948663/KF948664,KF948819/KF948820,KF949007/KF949008 |
|  | MSB193446 |  | 59.267 | -135.603 | KF948596,KF948665/KF948666,KF948821/KF948822,KF949009/KF949010 |
|  | MSB193447 |  | 59.267 | -135.603 | KF948597,KF948667/KF948668,KF948823/KF948824,KF949011/KF949012 |
|  | MSB193544 |  | 59.218 | -135.448 | KF948552, – / – ,KF948825/KF948826,KF949013/KF949014 |
|  | MSB195072 |  | 59.267 | -135.600 | KF948598, – / – ,KF948827/KF948828, – / – |
|  | MSB195212 |  | 59.246 | -135.175 | KF948600, – / – ,KF948831/KF948832,KF949015/KF949016 |
|  | MSB195220 |  | 59.300 | -135.704 | KF948601, – / – ,KF948833/KF948834,KF949017/KF949018 |
|  | UAM20577 |  | 59.433 | -135.950 | KF948614,KF948689/KF948690,KF948871/KF948872,KF949045/KF949046 |
|  | UAM31114 |  | 59.575 | -136.157 | **AF187217**, – / – , – / – , – / – |
|  | UAM52711 |  | 59.262 | -135.560 | KF964341,KF948749/KF948750,KF948947/KF948948,KF949119/KF949120 |
|  | UAM64491 |  | 59.286 | -136.108 | KF948576, – / – ,KF948963/KF948964, – / – |
|  | UAM64609 |  | 59.415 | -136.062 | KF948632,KF948763/KF948764,KF948965/KF948966, – / – |
|  | UAM64616 |  | 59.415 | -136.062 | KF948633,KF948765/KF948766,KF948967/KF948968,KF949133/KF949134 |
|  | UAM64624 |  | 59.366 | -135.798 | KF948545,KF948767/KF948768,KF948969/KF948970,KF949135/KF949136 |
|  | UAM68284 |  | 59.163 | -135.778 | KF948636,KF948773/KF948774,KF948975/KF948976,KF949141/KF949142 |
|  | UAM3553 | Alaska - interior | 61.500 | -142.833 | **AF187216**, – / – , – / – , – / – |
|  | UAM57777 |  | 61.318 | -144.235 | KF948628,KF948751/KF948752,KF948949/KF948950,KF949121/KF949122 |
|  | MSB148983 | Alaska - Island | 56.642 | -133.701 | KF948546,KF948647/KF948648,KF948793/KF948794,KF948991/KF948992 |
|  | MSB149385 |  | 56.668 | -134.266 | KF948547, – / – , – / – , – / – |
|  | MSB221332 |  | 55.488 | -133.631 | KF948602, – / – , – / – , – / – |
|  | MSB221340 |  | 55.488 | -133.631 | KF948603,KF948669/KF948670,KF948835/KF948836,KF949019/KF949020 |
|  | MSB221446 |  | 55.469 | -133.426 | KF948553,KF948671/KF948672,KF948837/KF948838,KF949021/KF949022 |
|  | MSB221498 |  | 55.469 | -133.426 | KF948604, – / – , – / – , – / – |
|  | MSB221521 |  | 55.469 | -133.426 | KF948605, – / – , – / – , – / – |
|  | MSB221545 |  | 55.469 | -133.426 | KF948606, – / – ,KF948839/KF948840, – / – |
|  | UAM20507 |  | 58.067 | -135.233 | **AF187211**, – / – ,KF948867/KF948868,KF949041/KF949042 |
|  | UAM20611 |  | 56.417 | -132.833 | KF948544, – / – ,KF948873/KF948874,KF949047/KF949048 |
|  | UAM20918 |  | 56.867 | -133.317 | **AF187208** , – / – , – / – , – / – |
|  | UAM20919 |  | 56.867 | -133.317 | KF948554, – / – , – / – , – / – |
|  | UAM22912 |  | 56.410 | -134.033 | **AF187224** ,KF948691/KF948692, – / – , – / – |
|  | UAM22913 |  | 56.410 | -134.033 | KF948555, – / – ,KF948875/KF948876,KF949049/KF949050 |
|  | UAM22918 |  | 56.583 | -132.833 | KF948556,KF948693/KF948694,KF948877/KF948878,KF949051/KF949052 |
|  | UAM22920 |  | 55.918 | -134.321 | KF964335, – / – , – / – , – / – |
|  | UAM22921 |  | 55.918 | -134.321 | **AF187223** , – / – , – / – , – / – |
|  | UAM23063 |  | 56.350 | -132.333 | KF948536,KF948695/KF948696,KF948879/KF948880,KF949053/KF949054 |
|  | UAM23410 |  | 55.918 | -134.321 | **AF187222** , – / – , – / – , – / – |
|  | UAM23455 |  | 55.502 | -131.028 | KF964344, – / – , – / – , – / – |
|  | UAM23606 |  | 55.918 | -134.321 | **AF187213** ,KF948697/KF948698,KF948883/KF948884,KF949057/KF949058 |
|  | UAM23659 |  | 55.452 | -133.660 | KF948615, – / – ,KF948885/KF948886,KF949059/KF949060 |
|  | UAM23726 |  | 54.767 | -132.183 | KF948616, – / – , – / – , – / – |
|  | UAM23727 |  | 54.767 | -132.183 | KF948557, – / – , – / – , – / – |
|  | UAM23728 |  | 54.783 | -132.867 | **AF187206** ,KF948699/KF948700,KF948887/KF948888,KF949061/KF949062 |
|  | UAM23804 |  | 55.767 | -131.017 | **AF187212**, – / – , – / – , – / – |
|  | UAM23871 |  | 56.975 | -133.941 | **AF187227** , – / – , – / – , – / – |
|  | UAM23943 |  | 55.817 | -131.367 | KF964336,KF948703/KF948704,KF948891/KF948892,KF949065/KF949066 |
|  | UAM23944 |  | 55.817 | -131.367 | KF948617,KF948705/KF948706,KF948893/KF948894,KF949067/KF949068 |
|  | UAM30504 |  | 55.415 | -131.696 | KF948618, – / – , – / – , – / – |
|  | UAM30506 |  | 56.150 | -133.350 | KF948559, – / – , – / – , – / – |
|  | UAM30507 |  | 56.150 | -133.350 | KF948560,KF948709/KF948710,KF948899/KF948900,KF949073/KF949074 |
|  | UAM30508 |  | 56.150 | -133.350 | KF948561,KF948711/KF948712,KF948901/KF948902,KF949075/KF949076 |
|  | UAM30721 |  | 56.150 | -133.350 | KF948619, – / – , – / – , – / – |
|  | UAM31755 |  | 55.875 | -133.842 | KF948542,KF948713/KF948714,KF948903/KF948904,KF949077/KF949078 |
|  | UAM31784 |  | 55.875 | -133.842 | **AF187218**, – / – , – / – , – / – |
|  | UAM31785 |  | 55.875 | -133.842 | KF948537, – / – , – / – , – / – |
|  | UAM31786 |  | 55.875 | -133.842 | **AF187214**, – / – , – / – , – / – |
|  | UAM31787 |  | 55.875 | -133.842 | KF948543, – / – , – / – , – / – |
|  | UAM31788 |  | 55.875 | -133.842 | KF948538,KF948715/KF948716,KF948905/KF948906,KF949079/KF949080 |
|  | UAM31826 |  | 55.100 | -132.833 | **AF187230**, – / – , – / – , – / – |
|  | UAM32929 |  | 57.817 | -136.150 | **AF187205**, – / – , – / – , – / – |
|  | UAM32932 |  | 57.817 | -136.150 | KF964338, – / – , – / – , – / – |
|  | UAM34288 |  | 56.174 | -133.369 | KF948621, – / – , – / – , – / – |
|  | UAM36269 |  | 58.079 | -135.478 | **AF187219**,KF948729/KF948730,KF948921/KF948922,KF949093/KF949094 |
|  | UAM36579 |  | 56.167 | -133.317 | **AF187215**,KF948731/KF948732,KF948923/KF948924,KF949095/KF949096 |
|  | UAM41767 |  | 55.461 | -132.692 | **AF187207**, – / – , – / – , – / – |
|  | UAM41768 |  | 55.283 | -133.307 | **AF187226**,KF948733/KF948734,KF948925/KF948926,KF949097/KF949098 |
|  | UAM42372 |  | 54.744 | -132.771 | KF948622, – / – , – / – , – / – |
|  | UAM42375 |  | 56.417 | -132.833 | KF948539,KF948735/KF948736,KF948927/KF948928,KF949099/KF949100 |
|  | UAM42376 |  | 56.321 | -134.072 | KF948565, – / – , – / – , – / – |
|  | UAM42377 |  | 56.583 | -134.000 | KF948640, – / – , – / – , – / – |
|  | UAM42380 |  | 54.821 | -133.521 | KF948566,KF948737/KF948738,KF948929/KF948930,KF949101/KF949102 |
|  | UAM42381 |  | 54.821 | -133.521 | **AF187221**,KF948739/KF948740, – / – ,KF949103/KF949104 |
|  | UAM42385 |  | 55.919 | -133.685 | KF948567, – / – , – / – , – / – |
|  | UAM42392 |  | 55.267 | -133.272 | KF948623, – / – , – / – , – / – |
|  | UAM42393 |  | 55.267 | -133.272 | KF948568, – / – , – / – , – / – |
|  | UAM42429 |  | 55.216 | -133.138 | KF948569, – / – , – / – , – / – |
|  | UAM42727 |  | 54.821 | -133.521 | KF948624, – / – , – / – , – / – |
|  | UAM42728 |  | 54.821 | -133.521 | KF948581, – / – ,KF948931/KF948932, – / – |
|  | UAM43227 |  | 55.267 | -133.272 | KF948582, – / – , – / – , – / – |
|  | UAM49638 |  | 55.283 | -133.307 | KF948578,KF948745/KF948746,KF948943/KF948944,KF949115/KF949116 |
|  | UAM49658 |  | 54.807 | -132.769 | KF948579,KF948747/KF948748,KF948945/KF948946,KF949117/KF949118 |
|  | UAM49660 |  | 54.807 | -132.769 | KF948627, – / – , – / – , – / – |
|  | UAM51686 |  | 57.958 | -134.307 | KF964342, – / – , – / – , – / – |
|  | UAM52256 |  | 55.950 | -133.383 | **AF187209**, – / – , – / – , – / – |
|  | UAM52262 |  | 55.900 | -133.333 | **AF187220**, – / – , – / – , – / – |
|  | UAM62899 |  | 56.233 | -132.133 | KF948540,KF948755/KF948756,KF948953/KF948954,KF949125/KF949126 |
|  | UAM70243 |  | 54.888 | -132.366 | KF948572,KF948775/KF948776,KF948977/KF948978,KF949143/KF949144 |
|  | UAM72153 |  | 56.318 | -132.286 | KF948541, – / – , – / – , – / – |
|  | UAM97289 |  | 55.344 | -131.498 | KF948575, – / – , – / – , – / – |
|  | MSB156999 | Alaska - Juneau | 58.343 | -134.640 | KF948550,KF948653/KF948654,KF948803/KF948804,KF948995/KF948996 |
|  | UAM74134 | Alaska - southeast | 57.367 | -133.467 | KF948573,KF948779/KF948780,KF948981/KF948982,KF949147/KF949148 |
|  | UAM74283 |  | 57.367 | -133.467 | KF948638, – / – , – / – , – / – |
|  | UAM74413 |  | 57.367 | -133.467 | KF948639,KF948781/KF948782,KF948983/KF948984,KF949149/KF949150 |
|  | UAM34297 | Yukon - south | 60.400 | -137.050 | **AF187228** ,KF948725/KF948726,KF948915/KF948916,KF949089/KF949090 |
|  | UAM34298 |  | 60.400 | -137.050 | **AF187229**,KF948727/KF948728,KF948917/KF948918,KF949091/KF949092 |
| Northern (N) | UAM34593 | Alaska - Glacier Bay | 65.228 | -144.500 | **AF187190**, – / – , – / – , – / – |
|  | UAM34594 |  | 65.228 | -144.500 | **AF187189**, – / – ,KF948919/KF948920, – / – |
|  | MSB195109 | Alaska - Haines | 59.267 | -135.600 | KF948599, – / – ,KF948829/KF948830, – / – |
|  | UAM20576 |  | 59.433 | -135.950 | KF948613,KF948687/KF948688,KF948869/KF948870,KF949043/KF949044 |
|  | UAM31113 |  | 59.575 | -136.157 | **AF187202**, – / – , – / – , – / – |
|  | UAM48471 |  | 59.500 | -135.354 | KF964340,KF948743/KF948744,KF948939/KF948940,KF949111/KF949112 |
|  | UAM64427 |  | 59.623 | -136.089 | KF948630,KF948757/KF948758,KF948955/KF948956,KF949127/KF949128 |
|  | UAM64428 |  | 59.623 | -136.089 | KF948570,KF948759/KF948760,KF948957/KF948958,KF949129/KF949130 |
|  | UAM64429 |  | 59.623 | -136.089 | KF948631, – / – ,KF948959/KF948960, – / – |
|  | UAM64442 |  | 59.623 | -136.089 | KF948571,KF948761/KF948762,KF948961/KF948962,KF949131/KF949132 |
|  | UAM70791 |  | 59.540 | -136.106 | KF948637,KF948777/KF948778,KF948979/KF948980,KF949145/KF949146 |
|  | UAM60319 | Alaska - interior | 65.303 | -142.037 | KF948629,KF948753/KF948754,KF948951/KF948952,KF949123/KF949124 |
|  | UAM65747 |  | 63.699 | -142.250 | KF948634,KF948769/KF948770,KF948971/KF948972,KF949137/KF949138 |
|  | UAM32822 | Alaska - Juneau | 58.183 | -133.317 | **AF187203**, – / – , – / – , – / – |
|  | UAM34292 |  | 58.533 | -133.683 | KF948563,KF948719/KF948720,KF948909/KF948910,KF949083/KF949084 |
|  | UAM34293 |  | 58.533 | -133.683 | KF948564,KF948721/KF948722,KF948911/KF948912,KF949085/KF949086 |
|  | UAM34294 |  | 58.533 | -133.683 | KF964337,KF948723/KF948724,KF948913/KF948914,KF949087/KF949088 |
|  | UAM75497 |  | 58.307 | -134.417 | KF948574,KF948783/KF948784,KF948985/KF948986,KF949151/KF949152 |
|  | UAM23496 | Alaska - southeast | 56.083 | -131.086 | **AF187191**, – / – ,KF948881/KF948882,KF949055/KF949056 |
|  | UAM23552 |  | 56.083 | -131.086 | KF964339, – / – , – / – , – / – |
|  | UAM23751 |  | 55.750 | -132.183 | **AF187198** , – / – , – / – , – / – |
|  | UAM23752 |  | 55.750 | -132.183 | KF964343,KF948701/KF948702,KF948889/KF948890,KF949063/KF949064 |
|  | UAM23883 |  | 55.767 | -130.883 | **AF187199**, – / – , – / – , – / – |
|  | UAM30495 |  | 55.750 | -132.183 | KF948558,KF948707/KF948708,KF948895/KF948896,KF949069/KF949070 |
|  | UAM30496 |  | 57.008 | -132.983 | **AF187193**, – / – , – / – , – / – |
|  | UAM30497 |  | 57.008 | -132.983 | **AF187201**, – / – , – / – , – / – |
|  | UAM34284 |  | 54.944 | -130.334 | KF948562,KF948717/KF948718,KF948907/KF948908,KF949081/KF949082 |
|  | UAM34285 |  | 54.944 | -130.334 | KF948620, – / – , – / – , – / – |
|  | UAM34286 |  | 54.944 | -130.334 | **AF187194**, – / – , – / – , – / – |
|  | UAM48474 |  | 56.027 | -130.071 | **AF187200**, – / – , – / – , – / – |
|  | UAM76246 |  | 55.767 | -130.883 | **AF187197**, – / – , – / – , – / – |
|  | UAM48463 | Alaska - White Pass | 59.614 | -135.167 | **AF187192** , – / – , – / – , – / – |
|  | UAM48465 |  | 59.614 | -135.167 | KF948625,KF948741/KF948742,KF948933/KF948934,KF949105/KF949106 |
|  | UAM48466 |  | 59.614 | -135.167 | KF948580, – / – ,KF948935/KF948936,KF949107/KF949108 |
|  | UAM48467 |  | 59.614 | -135.167 | KF948626, – / – ,KF948937/KF948938,KF949109/KF949110 |
|  | MSB155622 | British Columbia | 57.837 | -131.390 | KF948549, – / – , – / – , – / – |
|  | MSB158060 |  | 58.045 | -129.955 | KF948551, – / – ,KF948805/KF948806, – / – |
|  | MSB158239 |  | 56.496 | -129.425 | KF948535, – / – , – / – , – / – |
|  | UAM52714 |  | 57.866 | -131.284 | **AF187204**, – / – , – / – , – / – |
|  | UAM52715 |  | 57.855 | -131.369 | **AF187195**, – / – , – / – , – / – |
|  | UAM68182 |  | 59.529 | -134.908 | KF948635,KF948771/KF948772,KF948973/KF948974,KF949139/KF949140 |
|  | MSB144359 | Yukon | 60.065 | -128.619 | KF948583,KF948641/KF948642,KF948787/KF948788, – / – |
|  | MSB144391 |  | 60.065 | -128.619 | KF948584,KF948643/KF948644,KF948789/KF948790,KF948987/KF948988 |
|  | MSB144481 |  | 63.841 | -135.461 | KF948585,KF948645/KF948646,KF948791/KF948792,KF948989/KF948990 |
|  | MSB149145 |  | 61.996 | -132.609 | KF948586, – / – ,KF948795/KF948796, – / – |
|  | MSB149156 |  | 61.996 | -132.609 | KF948587, – / – ,KF948797/KF948798, – / – |
| North Pacific Coast (NPC) | UAM48484 | British Columbia | 59.734 | -133.667 | **AF187196** , – / – ,KF948941/KF948942,KF949113/KF949114 |
|  | UAM52717 |  | 59.734 | -133.667 | KF948577, – / – , – / – , – / – |
|  | UAM67039 |  | 50.817 | -118.967 | **AF187184**, – / – , – / – , – / – |
|  | UAM67078 |  | 50.817 | -120.467 | **AF187185**, – / – , – / – , – / – |
|  | UAM69491 |  | 50.817 | -118.967 | **AF187187**, – / – , – / – , – / – |
|  | UAM69492 |  | 50.817 | -118.967 | **AF187188**, – / – , – / – , – / – |
|  | UAM50787 | Oregon | 44.329 | -123.720 | **AF187183**, – / – , – / – , – / – |
|  | UAM64165 |  | 44.524 | -123.861 | **AF187182**, – / – , – / – , – / – |
|  | MSB43445 | Washington | 47.223 | -120.993 | **AF187180**,KF948679/KF948680,KF948847/KF948848, – / – |
|  | MSB80232 |  | 48.200 | -121.917 | KF948612,KF948685/KF948686,KF948863/KF948864,KF949037/KF949038 |
|  | UAM41789 |  | 47.000 | -120.950 | **AF187181**, – / – , – / – , – / – |
| Southern (S1) | MSB50495 | Arizona | 34.125 | -110.043 | **AF187164**, – / – ,KF948849/KF948850,KF949029/KF949030 |
|  | MSB53559 |  | 33.795 | -109.419 | **AF187165**, – / – , – / – , – / – |
|  | MSB88783 |  | 32.669 | -109.881 | – , – / – ,KF948865/KF948866,KF949039/KF949040 |
|  | MSB155126 | Colorado | 37.239 | -107.759 | KF948548, – / – ,KF948801/KF948802, – / – |
|  | MSB43689 | New Mexico | 35.813 | -106.523 | **AF187166**, – / – , – / – , – / – |
|  | MSB50701 |  | 35.267 | -106.199 | **AF187163**,KF948681/KF948682,KF948851/KF948852,KF949031/KF949032 |
| Southern (S2) | UAM41634 | Colorado | 38.529 | -106.325 | **AF187169**, – / – , – / – , – / – |
|  | UAM43522 |  | 38.529 | -106.325 | **AF187167**, – / – , – / – , – / – |
|  | UAM43523 |  | 38.529 | -106.325 | **AF187170**, – / – , – / – , – / – |
|  | UAM43524 |  | 38.529 | -106.325 | **AF187168**, – / – , – / – , – / – |
|  | MSB155085 | Wyoming | 41.037 | -110.404 | KF948588,KF948651/KF948652,KF948799/KF948800,KF948993/KF948994 |
|  | UAM69038 |  | 41.000 | -107.000 | **AF187171**, – / – , – / – , – / – |
| *M. montanus* | MSB121523 |  |  |  | KF948532, – / – , – / – , – / – |
| *M. montanus* |  |  |  |  | **AF119280**, – / – , – / – , – / – |
| *M. pennsylvanicus* | MSB110998 |  |  |  | KF948531, – / – , – / – , – / – |
| *M. pennsylvanicus* | MSB149290 |  |  |  | – ,KF948649/KF948650, – / – , – / – |
| *M. pennsylvanicus* |  |  |  |  | **AF119279**, – / – , – / – , – / – |
| *M. pennsylvanicus* |  |  |  |  | – , – / – , – / – ,**AY241463**/ – |
