# Supplementary material for: Phylogeographic structure in long‐tailed voles (Rodentia: Arvicolinae) belies the complex Pleistocene history of isolation, divergence, and recolonization of Northwest North America's fauna
Source: Ecol Evol. 2016 Aug 29;6(18):6633–47. doi: 10.1002/ece3.2393 (PMC5058534; doi:10.1002/ece3.2393)
Supplement: Supplementary file 2 — Figure S1. Dated Bayesian cytb trees. Figure S2. Sampling localities for species distribution models. Figure S3. Phased Bayesian gene trees for (A) ETS2, (B) FGB, and (C) Rag1 nuclear loci with posterior probabilities of ≥0.95 represented with open circles on branches of the solid consensus tree. Figure S4. Phased nuclear haplotype distribution in Northern and Island clades of M. longicaudus. Table S1. Primer list and PCR annealing temperatures. Table S2. Diversity indices, expansion statistics and models of evolution. Table S3. Between group net genetic distance. Table S4. Locality abbreviations. Table S5. Island and Northern cytb clade populations near the geographic regions of contact (Haines and Juneau, Alaska). Table S6. Bayesian migration estimates for Southeast Alaska populations determined in bayesass for M. longicaudus. Table S7. Cyt b and phased multilocus divergence date estimates. Data S1. Cyt b data ‐ Methods and Results. [file ECE3-6-6633-s002.docx]

Supplemental Figure 1. Dated Bayesian cyt*b* trees. Bayesian Posterior probability ≥0.95 represented with open circles and Maximum Likelihood bootstraps of ≥0.7 with asterisks are shown on branches. Vertical gray bars represent the LIG (left) and LGM (right). Geographic locations for supported intralineage clades are provided. NPC = North Pacific Coast; COP = Colorado Plateau. See Supplemental Table 4 for abbreviations.


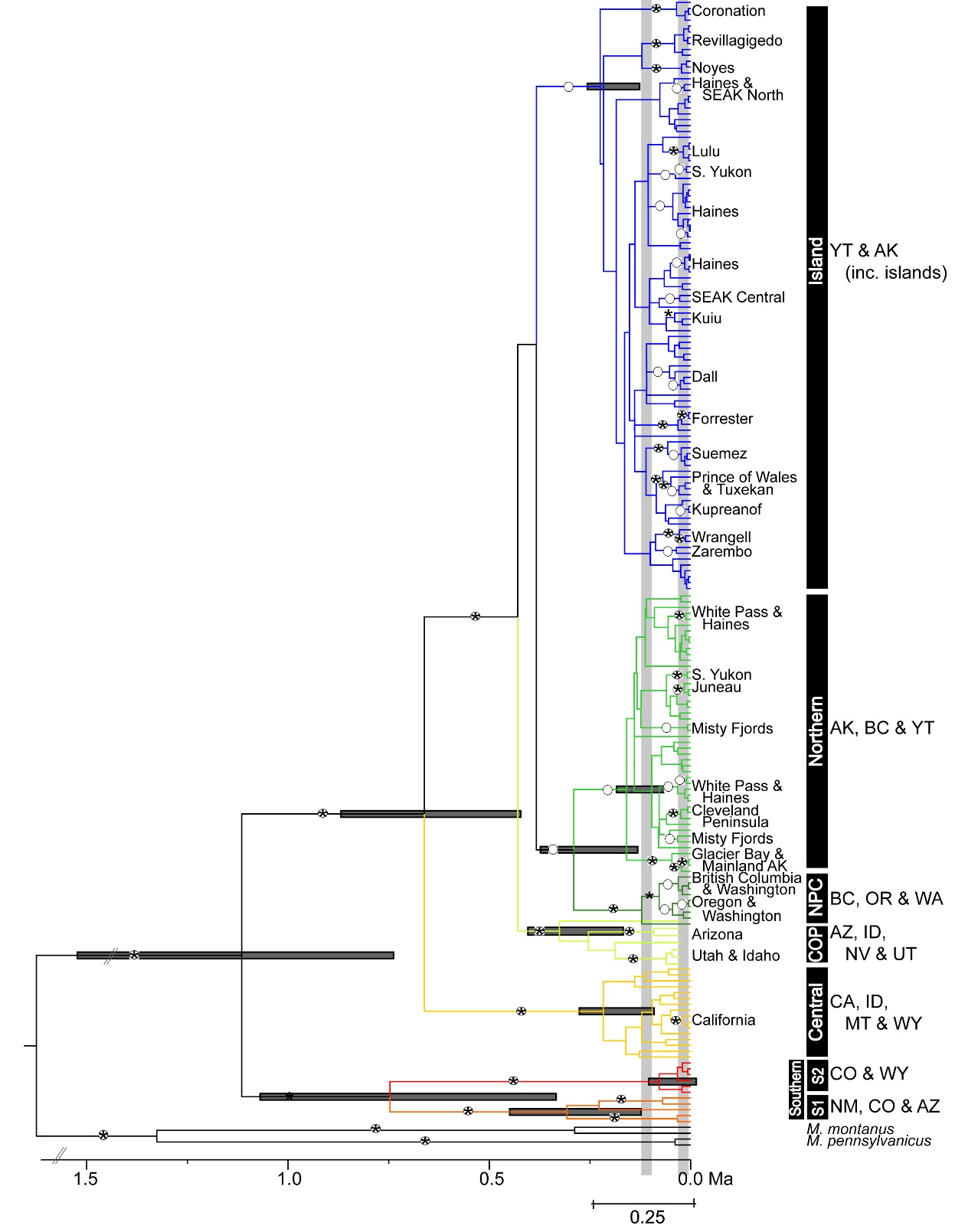


Supplemental Figure 2. Sampling localities for species distribution models.


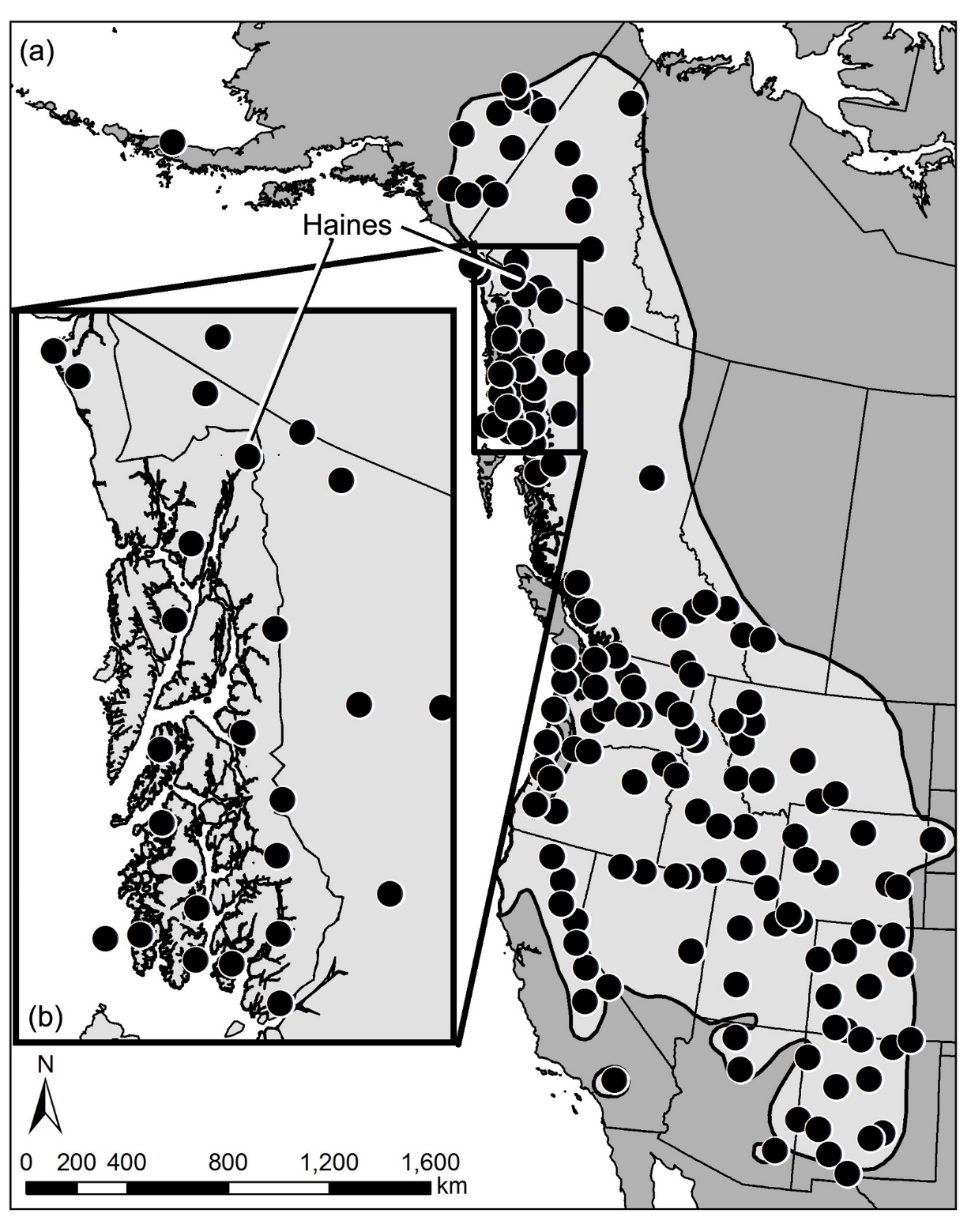


Supplemental Figure 3. Phased Bayesian gene trees for (a) ETS2, (b) FGB, and (c) Rag1 nuclear loci with posterior probabilities of ≥0.95 represented with open circles on branches of the solid consensus tree. Black dots are Haines individuals. Blue = Island, middle green = Northern, dark green = NPC, light green = COP, gold = Central, orange = S1, and red = S2. Geographic locations (Supplemental Table 4) for supported lineages are provided.


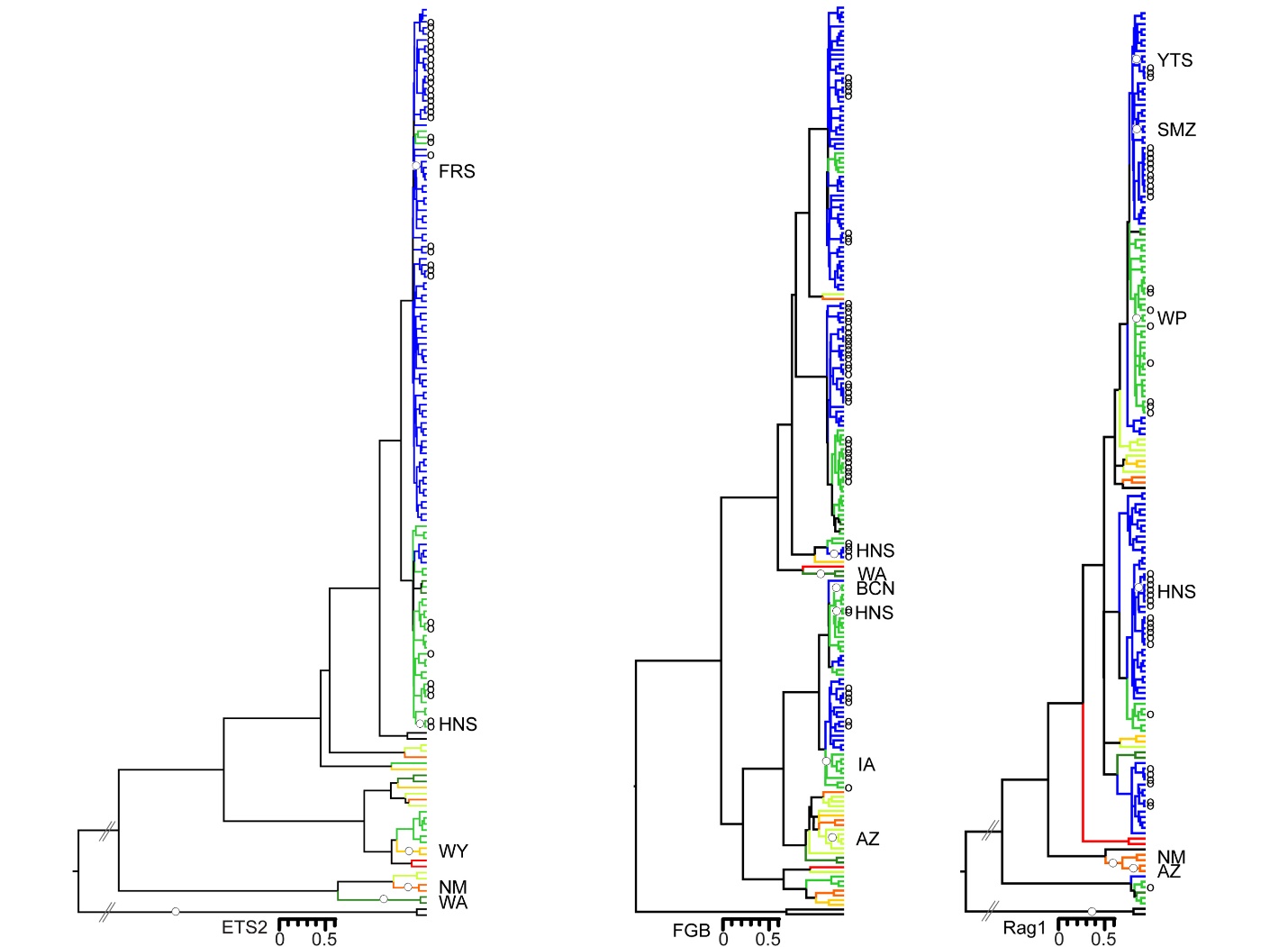


Supplemental Figure 4. Phased nuclear haplotype distribution in Northern and Island clades of *M. longicaudus*. (a) ETS2, (b) FGB, and (c) Rag1. Thick black line delimits Island (left) and Northern (right) lineages with sympatry indicated with black sample locations and haplotype variety indicated with adjoining lines. Each color within a locus represents a unique haplotype.


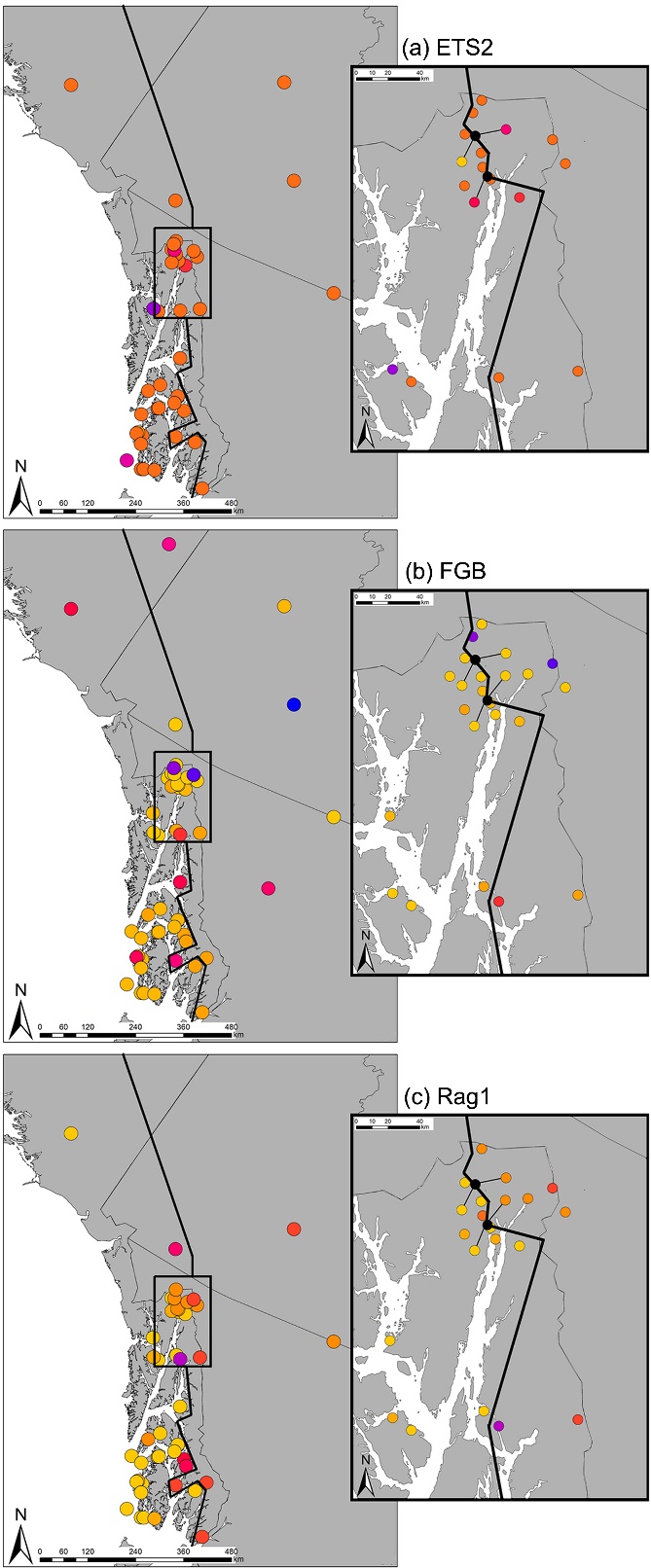


Supplemental Table 1. Primer list and PCR annealing temperatures. Primers used for amplification and sequencing mtDNA Cytochrome B (cyt*b*), and nuclear loci Protein C-est-2 (ETS2), β-fibrinogen (FGB), and Recombination Activating Protein 1 (Rag1) in *M. longicaudus* and outgroup taxa, with annealing temperatures (^o^C) indicated in parentheses.

| **Primer** | **Sequence (5'-3')** | **Reference** |
| --- | --- | --- |
| **cyt*b* (50)** | |  |
| L14724 |  | (Irwin *et al.* 1991; Kocher & White 1989) |
| Vole 14 |  | (Conroy & Cook 1999) |
|  |  |  |
| **ETS2 (63)** | |  |
| ETS2F |  | (Lyons *et al.* 1997) |
| ETS2R |  | (Lyons *et al.* 1997) |
|  |  |  |
| **FGB (65)** | |  |
| MSB_MFGBF | CGTTTGGATTGGCGGAGTGG | This study, modified from Matocq *et al*. (2007) |
| MSB_MFGBR | GCACGTACGACAGGGACAACG | This study, modified from Matocq *et al*. (2007) |
|  |  |  |
| **Rag1 (60)** | |  |
| MSB_Rag1F | GCAGTCTCCTTTAGTTCCAGAC | This study, modified from Steppan *et al*.(2004) |
| MSB_Rag1R | CCAACAGGAACAACGTCAAGC | This study, modified from Steppan *et al*.(2004) |

Supplemental Table 2. Diversity indices, expansion statistics and models of evolution. n = haploid sample size; L = length of sequence; *S* = variable sites; *h* = #haplotypes; *Hd* = haplotype diversity; *π* = nucleotide diversity; *D* = Tajima's D; *Fs* = Fu's FS; *R2* = Ramos-Onsin's R2; Model = model of evolution as selected by ModelTest. Bold values are significant at p<0.05 (p<0.02 for FS).

| **Group** | **Gene** | **n** | **L** | ***S*** | ***h*** | ***Hd*** | ***π*** | ***D*** | ***Fs* (p<.02)** | ***R_2_*** | **Model of Evolution** |
| --- | --- | --- | --- | --- | --- | --- | --- | --- | --- | --- | --- |
| All Samples | Cytb | 196 | 1143 | 175 | 127 | 0.990 | 0.01409 | **-0.100** | **-0.776** | **0.082** | TrN+I+G |
|  | ETS2 | 80 | 733 | – | – | – | – | – | – | – | GTR+I+G |
|  | FGB | 110 | 600 | – | – | – | – | – | – | – | HKY+G |
|  | Rag1 | 182 | 963 | – | – | – | – | – | – | – | HKY |
| No Outgroups | Cytb | 192 | 1143 | 133 | 123 | 0.990 | 0.01137 | **-0.075** | **-0.656** | **0.082** |  |
|  | ETS2 | 78 | 733 | 4 | 5 | 0.169 | 0.00026 | **-0.005** | **-0.143** | **0.097** |  |
|  | FGB | 108 | 600 | 13 | 13 | 0.126 | 0.00024 | **-0.005** | **-0.252** | **0.091** |  |
|  | Rag1 | 89 | 963 | 10 | 11 | 0.162 | 0.00020 | **-0.028** | **-0.186** | **0.095** |  |
| Central | Cytb | 16 | 1143 | 37 | 14 | 0.975 | 0.00761 | -0.073 | **0.031** | **0.140** | HKY+I |
|  | ETS2 | 9 | 733 | 3 | 4 | 0.654 | 0.00117 | -0.014 | 0.187 | **0.167** | GTR+I |
|  | FGB | 9 | 600 | 17 | 13 | 0.928 | 0.00793 | -0.105 | **0.055** | **0.138** | GTR+I |
|  | Rag1 | 8 | 963 | 3 | 4 | 0.350 | 0.00039 | **-0.022** | **0.266** | **0.190** | HKY |
| Colorado Plateau | Cytb | 7 | 1143 | 22 | 7 | 1.000 | 0.00877 | -0.055 | 0.503 | **0.190** |  |
|  | ETS2 | 2 | 733 | 12 | 2 | 0.667 | 0.01103 | -0.046 | 0.962 | **0.266** |  |
|  | FGB | 7 | 600 | 8 | 7 | 0.879 | 0.00354 | -0.043 | 0.179 | **0.163** |  |
|  | Rag1 | 3 | 963 | 1 | 2 | 0.333 | 0.00035 | -0.005 | 0.324 | **0.305** |  |
| Island | Cytb | 101 | 1143 | 70 | 61 | 0.983 | 0.00470 | **-0.079** | **-0.319** | **0.092** | HKY+I+G |
|  | ETS2 | 43 | 733 | 7 | 4 | 0.113 | 0.00044 | **-0.024** | -0.039 | 0.109 | HKY |
|  | FGB | 55 | 600 | 8 | 9 | 0.157 | 0.00028 | **-0.005** | -0.080 | **0.107** | HKY+I |
|  | Rag1 | 50 | 963 | 3 | 4 | 0.116 | 0.00013 | **-0.019** | **-0.117** | **0.110** | TrN |
| North | Cytb | 46 | 1143 | 53 | 29 | 0.961 | 0.00395 | **-0.094** | **-0.153** | **0.108** | HKY+I |
|  | ETS2 | 20 | 733 | 16 | 8 | 0.501 | 0.00043 | -0.073 | -0.087 | **0.113** | HKY+I |
|  | FGB | 29 | 600 | 5 | 7 | 0.728 | 0.00192 | -0.053 | -0.016 | **0.109** | GTR+I |
|  | Rag1 | 22 | 963 | 3 | 4 | 0.452 | 0.00052 | -0.018 | 0.090 | **0.130** | HKY |
| North Pacific Coast | Cytb | 11 | 1143 | 16 | 8 | 0.945 | 0.00511 | -0.063 | 0.169 | **0.163** |  |
|  | ETS2 | 2 | 733 | 11 | 3 | 0.833 | 0.00989 | -0.025 | 0.911 | **0.268** |  |
|  | FGB | 3 | 600 | 11 | 4 | 0.867 | 0.01002 | -0.046 | 0.493 | **0.213** |  |
|  | Rag1 | 2 | 963 | 0 | 1 | 0.000 | 0.00000 | – | – | – |  |
| South | Cytb | 11 | 1143 | 36 | 9 | 0.945 | 0.01378 | -0.090 | 0.330 | **0.156** | HKY+I |
|  | ETS2 | 2 | 733 | 17 | 3 | 0.833 | 0.01551 | -0.050 | 1.161 | **0.257** | HKY |
|  | FGB | 5 | 600 | 7 | 7 | 0.867 | 0.00501 | -0.056 | 0.192 | **0.179** | HKY+I |
|  | Rag1 | 4 | 963 | 4 | 5 | 0.893 | 0.00156 | -0.030 | 0.289 | **0.221** | HKY |

Supplemental Table 3. Between group net genetic distance. The number of base differences per site from estimation of net average between groups of sequences are shown. Standard error estimate(s) is above the diagonal. All ambiguous positions were removed for each sequence pair. Evolutionary analyses were conducted in MEGA5.

|  | Central | COP | Island | NPC | Northern | Southern | *M. pennsylvanicus* | *M. montanus* |
| --- | --- | --- | --- | --- | --- | --- | --- | --- |
| Central |  | 0.40% | 0.40% | 0.40% | 0.40% | 0.40% | 0.80% | 0.70% |
| COP | 2.30% |  | 0.30% | 0.20% | 0.10% | 0.40% | 0.80% | 0.80% |
| Island | 2.50% | 1.10% |  | 0.20% | 0.30% | 0.40% | 0.80% | 0.70% |
| NPC | 2.30% | 0.80% | 1.10% |  | 0.30% | 0.40% | 0.80% | 0.70% |
| Northern | 2.60% | 0.50% | 1.10% | 1.10% |  | 0.50% | 0.80% | 0.70% |
| Southern | 3.30% | 3.30% | 3.30% | 2.90% | 3.30% |  | 0.80% | 0.70% |
| *M. pennsylvanicus* | 10.20% | 10.10% | 10.00% | 9.80% | 10.00% | 9.90% |  | 0.60% |
| *M. montanus* | 8.70% | 9.00% | 8.50% | 8.40% | 8.80% | 7.70% | 4.70% |  |

Supplemental Table 4. Locality abbreviations. Standard state and province abbreviations apply for unlisted locations.

| **Region** | **Location** |
| --- | --- |
| Alaskan Islands | Chichagof (CGF) |
|  | Coronation (CRN) |
|  | Dall (DAL) |
|  | Forrester (FRS) |
|  | Kosciusko (KSC) |
|  | Kuiu (KUI) |
|  | Kupreanof (KRF) |
|  | Lulu (LUL) |
|  | Mitkof (MIT) |
|  | Noyes (NYS) |
|  | Orr (ORI) |
|  | Prince of Wales (POW) |
|  | Revillagigedo (REV) |
|  | Suemez (SMZ) |
|  | Sukkwan (SWN) |
|  | Tuxekan (TXN) |
|  | Warren (WRN) |
|  | Wrangell (WRG) |
|  | Zarembo (ZRB) |
| mainland | British Columbia (BC) |
|  | Mainland Southeast Alaska (MLSE) |
|  | Northern mainalnd Southeast (MLSEN) |
|  | Cleveland Peninsula (CP/MLCP) |
|  | Foggy Bay (FB/MLFB) |
|  | Glacier Bay (GB/MLGB) |
|  | Haines (HNS/MLHNS) |
|  | Interior Alaska (IAK/MLS) |
|  | Juneau (JNO/MLJNO) |
|  | Klukwan (KLU/MLKLU) |
|  | Misty Fjords (MFD/MLMFD) |
|  | Southeast Central (SEC AK/MLSEC) |
|  | Southeast North (SEN AK/MLSEN) |
|  | Southeast South (SES AK/MLSES) |
|  | Skagway (SKW/MLSKW) |
|  | Taiya River (TYR/MLTYR) |
|  | White Pass (WP/MLWP) |
|  | British Columbia - Central (BCC) |
|  | British Columbia - North (BCN) |
|  | British Columbia - South (BCS) |
|  | Washington (WA) |
|  | Yukon Territory - Central (YTC) |
|  | Yukon Territory - South (YTS) |

Supplemental Table 5. Island and Northern cyt*b* clade populations near the geographic regions of contact (Haines and Juneau, Alaska). Bayesian migration estimates determined in BayesAss. Non-migrants within each population are indicated in bold along the diagonal. Values are the proportion of migrant genes donated from source populations (columns) into sink populations (rows).

|  | **Migration rates into…** | | |  |  |  |
| --- | --- | --- | --- | --- | --- | --- |
| **From…** | **I-interior AK** | **I-Glacier Bay** | **I-Haines** | **I-Juneau** | **I-SE AK** | **I-Yukon** |
| **I-interior AK** | **0.682** | 0.014 | 0.025 | 0.014 | 0.014 | 0.014 |
| **I-Glacier Bay** | 0.015 | **0.682** | 0.026 | 0.015 | 0.014 | 0.015 |
| **I-Haines** | 0.008 | 0.008 | **0.830** | 0.008 | 0.008 | 0.008 |
| **I-Juneau** | 0.014 | 0.015 | 0.027 | **0.682** | 0.014 | 0.014 |
| **I-SE AK** | 0.013 | 0.013 | 0.046 | 0.013 | **0.680** | 0.013 |
| **I-Yukon** | 0.014 | 0.014 | 0.035 | 0.014 | 0.014 | **0.681** |
| **N-interior AK** | 0.014 | 0.014 | 0.029 | 0.014 | 0.014 | 0.014 |
| **N-Glacier Bay** | 0.014 | 0.014 | 0.018 | 0.014 | 0.014 | 0.014 |
| **N-Haines** | 0.012 | 0.012 | 0.074 | 0.012 | 0.011 | 0.012 |
| **N-Juneau** | 0.013 | 0.013 | 0.063 | 0.013 | 0.013 | 0.013 |
| **N-SE AK** | 0.010 | 0.010 | 0.030 | 0.010 | 0.010 | 0.010 |
| **N-Yukon** | 0.013 | 0.013 | 0.049 | 0.013 | 0.013 | 0.013 |

|  |  |  | **Migration rates into…** | | |  |
| --- | --- | --- | --- | --- | --- | --- |
| **N-interior AK** | **N-Glacier Bay** | **N-Haines** | **N-Juneau** | **N-SE AK** | **N-Yukon** | **From…** |
| 0.014 | 0.014 | 0.014 | 0.014 | 0.022 | 0.014 | **I-interior AK** |
| 0.015 | 0.015 | 0.015 | 0.015 | 0.015 | 0.015 | **I-Glacier Bay** |
| 0.008 | 0.008 | 0.008 | 0.008 | 0.009 | 0.008 | **I-Haines** |
| 0.014 | 0.015 | 0.015 | 0.015 | 0.016 | 0.015 | **I-Juneau** |
| 0.013 | 0.013 | 0.013 | 0.013 | 0.023 | 0.013 | **I-SE AK** |
| 0.014 | 0.014 | 0.014 | 0.014 | 0.017 | 0.014 | **I-Yukon** |
| **0.681** | 0.014 | 0.015 | 0.014 | 0.020 | 0.014 | **N-interior AK** |
| 0.014 | **0.682** | 0.015 | 0.014 | 0.025 | 0.015 | **N-Glacier Bay** |
| 0.011 | 0.012 | **0.681** | 0.011 | 0.026 | 0.012 | **N-Haines** |
| 0.013 | 0.013 | 0.013 | **0.680** | 0.019 | 0.013 | **N-Juneau** |
| 0.010 | 0.010 | 0.010 | 0.010 | **0.770** | 0.011 | **N-SE AK** |
| 0.013 | 0.013 | 0.013 | 0.013 | 0.022 | **0.680** | **N-Yukon** |

Supplemental Table 6 Bayesian migration estimates for Southeast Alaska populations determined in BayesAss for *M. longicaudus*. Non-migrants within each population are indicated in bold along the diagonal. Values are the proportion of migrant genes donated from source populations (columns) into sink populations (rows). Location abbreviations are in Supplemental Table 2 and I = Island and N = Northern clades.

|  | | **Migration rates into…** | | | | | |  |  | |  | |  | |  |  | |  | |  | |  | |  |  | | |  |  | |  |  |  |  |  |  |  |
| --- | --- | --- | --- | --- | --- | --- | --- | --- | --- | --- | --- | --- | --- | --- | --- | --- | --- | --- | --- | --- | --- | --- | --- | --- | --- | --- | --- | --- | --- | --- | --- | --- | --- | --- | --- | --- | --- |
| **From…** | | **I_CGF** | | **I_CRN** | | **I_DAL** | | **I_FST** | **I_KRF** | | **I_KSC** | | **I_KUI** | | **I_LUL** | **I_MIT** | | **I_MLGB** | | **I_MLHNS** | | **I_MLJNO** | | **I_MLS** | **I_MLSEC** | | | **I_MLSEN** | **I_NYS** | | **I_ORI** | **I_POW** | **I_REV** | **I_SMZ** | **I_SWN** | **I_TXN** | **I_WRG** |
| **I_CGF** | | **0.6773** | | 0.0094 | | 0.0092 | | 0.0093 | 0.0094 | | 0.0094 | | 0.0092 | | 0.0091 | 0.0089 | | 0.0089 | | 0.0106 | | 0.0089 | | 0.0090 | 0.0090 | | | 0.0088 | 0.0093 | | 0.0089 | 0.0096 | 0.0097 | 0.0092 | 0.0090 | 0.0089 | 0.0091 |
| **I_CRN** | | 0.0092 | | **0.6779** | | 0.0093 | | 0.0094 | 0.0094 | | 0.0094 | | 0.0091 | | 0.0091 | 0.0089 | | 0.0088 | | 0.0106 | | 0.0089 | | 0.0089 | 0.0090 | | | 0.0088 | 0.0093 | | 0.0089 | 0.0096 | 0.0100 | 0.0091 | 0.0090 | 0.0089 | 0.0091 |
| **I_DAL** | | 0.0093 | | 0.0096 | | **0.6769** | | 0.0095 | 0.0094 | | 0.0095 | | 0.0091 | | 0.0091 | 0.0089 | | 0.0087 | | 0.0111 | | 0.0089 | | 0.0088 | 0.0089 | | | 0.0088 | 0.0094 | | 0.0089 | 0.0098 | 0.0102 | 0.0090 | 0.0089 | 0.0089 | 0.0090 |
| **I_FST** | | 0.0092 | | 0.0096 | | 0.0093 | | **0.6772** | 0.0094 | | 0.0094 | | 0.0092 | | 0.0092 | 0.0089 | | 0.0090 | | 0.0103 | | 0.0090 | | 0.0089 | 0.0091 | | | 0.0088 | 0.0093 | | 0.0090 | 0.0097 | 0.0099 | 0.0092 | 0.0090 | 0.0090 | 0.0091 |
| **I_KRF** | | 0.0093 | | 0.0094 | | 0.0092 | | 0.0092 | **0.6774** | | 0.0094 | | 0.0091 | | 0.0092 | 0.0089 | | 0.0089 | | 0.0103 | | 0.0090 | | 0.0090 | 0.0090 | | | 0.0089 | 0.0094 | | 0.0090 | 0.0097 | 0.0098 | 0.0092 | 0.0090 | 0.0090 | 0.0091 |
| **I_KSC** | | 0.0093 | | 0.0096 | | 0.0091 | | 0.0094 | 0.0095 | | **0.6774** | | 0.0091 | | 0.0091 | 0.0089 | | 0.0088 | | 0.0110 | | 0.0089 | | 0.0088 | 0.0089 | | | 0.0088 | 0.0094 | | 0.0088 | 0.0099 | 0.0101 | 0.0091 | 0.0089 | 0.0089 | 0.0089 |
| **I_KUI** | | 0.0093 | | 0.0094 | | 0.0093 | | 0.0093 | 0.0095 | | 0.0094 | | **0.6770** | | 0.0091 | 0.0089 | | 0.0089 | | 0.0101 | | 0.0090 | | 0.0089 | 0.0091 | | | 0.0089 | 0.0094 | | 0.0090 | 0.0095 | 0.0098 | 0.0092 | 0.0090 | 0.0090 | 0.0091 |
| **I_LUL** | | 0.0093 | | 0.0095 | | 0.0092 | | 0.0094 | 0.0095 | | 0.0094 | | 0.0092 | | **0.6767** | 0.0090 | | 0.0089 | | 0.0104 | | 0.0090 | | 0.0089 | 0.0091 | | | 0.0089 | 0.0093 | | 0.0089 | 0.0096 | 0.0098 | 0.0092 | 0.0091 | 0.0089 | 0.0091 |
| **I_MIT** | | 0.0092 | | 0.0093 | | 0.0092 | | 0.0093 | 0.0093 | | 0.0094 | | 0.0092 | | 0.0092 | **0.6761** | | 0.0091 | | 0.0100 | | 0.0092 | | 0.0091 | 0.0092 | | | 0.0091 | 0.0093 | | 0.0091 | 0.0095 | 0.0095 | 0.0092 | 0.0092 | 0.0092 | 0.0092 |
| **I_MLGB** | | 0.0092 | | 0.0093 | | 0.0093 | | 0.0094 | 0.0093 | | 0.0093 | | 0.0092 | | 0.0092 | 0.0092 | | **0.6761** | | 0.0097 | | 0.0091 | | 0.0092 | 0.0092 | | | 0.0092 | 0.0093 | | 0.0091 | 0.0094 | 0.0096 | 0.0092 | 0.0092 | 0.0091 | 0.0092 |
| **I_MLHNS** | | 0.0095 | | 0.0097 | | 0.0090 | | 0.0095 | 0.0095 | | 0.0095 | | 0.0088 | | 0.0090 | 0.0087 | | 0.0086 | | **0.6821** | | 0.0087 | | 0.0086 | 0.0088 | | | 0.0084 | 0.0094 | | 0.0085 | 0.0100 | 0.0107 | 0.0090 | 0.0088 | 0.0085 | 0.0089 |
| **I_MLJNO** | | 0.0092 | | 0.0094 | | 0.0092 | | 0.0093 | 0.0093 | | 0.0093 | | 0.0092 | | 0.0092 | 0.0091 | | 0.0092 | | 0.0100 | | **0.6760** | | 0.0091 | 0.0092 | | | 0.0091 | 0.0093 | | 0.0091 | 0.0094 | 0.0096 | 0.0092 | 0.0092 | 0.0091 | 0.0092 |
| **I_MLS** | | 0.0093 | | 0.0094 | | 0.0093 | | 0.0093 | 0.0093 | | 0.0093 | | 0.0092 | | 0.0092 | 0.0091 | | 0.0092 | | 0.0095 | | 0.0092 | | **0.6761** | 0.0092 | | | 0.0092 | 0.0093 | | 0.0092 | 0.0093 | 0.0095 | 0.0092 | 0.0092 | 0.0092 | 0.0092 |
| **I_MLSEC** | | 0.0092 | | 0.0094 | | 0.0092 | | 0.0095 | 0.0094 | | 0.0095 | | 0.0091 | | 0.0092 | 0.0090 | | 0.0090 | | 0.0102 | | 0.0090 | | 0.0090 | **0.6763** | | | 0.0089 | 0.0093 | | 0.0090 | 0.0095 | 0.0099 | 0.0092 | 0.0090 | 0.0090 | 0.0091 |
| **I_MLSEN** | | 0.0093 | | 0.0093 | | 0.0093 | | 0.0093 | 0.0093 | | 0.0093 | | 0.0093 | | 0.0093 | 0.0092 | | 0.0092 | | 0.0094 | | 0.0092 | | 0.0092 | 0.0092 | | | **0.6762** | 0.0092 | | 0.0092 | 0.0093 | 0.0093 | 0.0093 | 0.0092 | 0.0093 | 0.0093 |
| **I_NYS** | | 0.0093 | | 0.0094 | | 0.0092 | | 0.0093 | 0.0094 | | 0.0095 | | 0.0092 | | 0.0092 | 0.0090 | | 0.0090 | | 0.0104 | | 0.0090 | | 0.0090 | 0.0091 | | | 0.0089 | **0.6767** | | 0.0090 | 0.0096 | 0.0097 | 0.0092 | 0.0090 | 0.0090 | 0.0091 |
| **I_ORI** | | 0.0093 | | 0.0093 | | 0.0093 | | 0.0093 | 0.0093 | | 0.0093 | | 0.0093 | | 0.0093 | 0.0092 | | 0.0092 | | 0.0093 | | 0.0092 | | 0.0092 | 0.0092 | | | 0.0092 | 0.0093 | | **0.6762** | 0.0093 | 0.0093 | 0.0093 | 0.0092 | 0.0092 | 0.0093 |
| **I_POW** | | 0.0093 | | 0.0096 | | 0.0092 | | 0.0095 | 0.0095 | | 0.0094 | | 0.0091 | | 0.0091 | 0.0087 | | 0.0087 | | 0.0113 | | 0.0088 | | 0.0087 | 0.0090 | | | 0.0087 | 0.0093 | | 0.0087 | **0.6783** | 0.0102 | 0.0090 | 0.0089 | 0.0087 | 0.0090 |
| **I_REV** | | 0.0094 | | 0.0096 | | 0.0091 | | 0.0095 | 0.0094 | | 0.0094 | | 0.0090 | | 0.0091 | 0.0087 | | 0.0088 | | 0.0107 | | 0.0087 | | 0.0088 | 0.0090 | | | 0.0086 | 0.0093 | | 0.0087 | 0.0095 | **0.6799** | 0.0091 | 0.0088 | 0.0087 | 0.0090 |
| **I_SMZ** | | 0.0093 | | 0.0095 | | 0.0093 | | 0.0094 | 0.0094 | | 0.0095 | | 0.0091 | | 0.0091 | 0.0089 | | 0.0088 | | 0.0108 | | 0.0089 | | 0.0088 | 0.0090 | | | 0.0088 | 0.0093 | | 0.0089 | 0.0098 | 0.0101 | **0.6768** | 0.0089 | 0.0089 | 0.0090 |
| **I_SWN** | | 0.0092 | | 0.0093 | | 0.0092 | | 0.0092 | 0.0093 | | 0.0093 | | 0.0093 | | 0.0093 | 0.0093 | | 0.0092 | | 0.0094 | | 0.0092 | | 0.0092 | 0.0092 | | | 0.0092 | 0.0093 | | 0.0092 | 0.0092 | 0.0093 | 0.0093 | **0.6762** | 0.0092 | 0.0092 |
| **I_TXN** | | 0.0092 | | 0.0093 | | 0.0092 | | 0.0092 | 0.0093 | | 0.0092 | | 0.0092 | | 0.0093 | 0.0092 | | 0.0092 | | 0.0093 | | 0.0092 | | 0.0092 | 0.0092 | | | 0.0092 | 0.0092 | | 0.0092 | 0.0093 | 0.0093 | 0.0092 | 0.0092 | **0.6762** | 0.0092 |
| **I_WRG** | | 0.0093 | | 0.0094 | | 0.0092 | | 0.0093 | 0.0093 | | 0.0094 | | 0.0093 | | 0.0092 | 0.0091 | | 0.0091 | | 0.0097 | | 0.0090 | | 0.0090 | 0.0091 | | | 0.0090 | 0.0093 | | 0.0091 | 0.0094 | 0.0097 | 0.0092 | 0.0090 | 0.0091 | **0.6765** |
| **I_WRN** | | 0.0093 | | 0.0096 | | 0.0091 | | 0.0094 | 0.0095 | | 0.0094 | | 0.0090 | | 0.0090 | 0.0087 | | 0.0087 | | 0.0113 | | 0.0088 | | 0.0087 | 0.0089 | | | 0.0086 | 0.0093 | | 0.0087 | 0.0099 | 0.0102 | 0.0090 | 0.0089 | 0.0087 | 0.0089 |
| **I_YTS** | | 0.0093 | | 0.0094 | | 0.0092 | | 0.0093 | 0.0093 | | 0.0094 | | 0.0092 | | 0.0092 | 0.0091 | | 0.0091 | | 0.0100 | | 0.0091 | | 0.0091 | 0.0091 | | | 0.0090 | 0.0093 | | 0.0091 | 0.0095 | 0.0096 | 0.0092 | 0.0091 | 0.0091 | 0.0092 |
| **I_ZRB** | | 0.0092 | | 0.0094 | | 0.0093 | | 0.0094 | 0.0094 | | 0.0093 | | 0.0091 | | 0.0092 | 0.0091 | | 0.0090 | | 0.0101 | | 0.0091 | | 0.0091 | 0.0091 | | | 0.0091 | 0.0093 | | 0.0091 | 0.0095 | 0.0097 | 0.0092 | 0.0091 | 0.0091 | 0.0092 |
| **N_MLCP** | | 0.0094 | | 0.0093 | | 0.0093 | | 0.0092 | 0.0094 | | 0.0094 | | 0.0092 | | 0.0092 | 0.0090 | | 0.0090 | | 0.0096 | | 0.0090 | | 0.0090 | 0.0091 | | | 0.0090 | 0.0093 | | 0.0091 | 0.0095 | 0.0096 | 0.0092 | 0.0091 | 0.0091 | 0.0091 |
| **N_MLGB** | | 0.0093 | | 0.0093 | | 0.0092 | | 0.0093 | 0.0093 | | 0.0093 | | 0.0092 | | 0.0092 | 0.0091 | | 0.0091 | | 0.0093 | | 0.0092 | | 0.0092 | 0.0092 | | | 0.0092 | 0.0093 | | 0.0092 | 0.0092 | 0.0093 | 0.0092 | 0.0092 | 0.0091 | 0.0092 |
| **N_MLHNS** | | 0.0092 | | 0.0092 | | 0.0093 | | 0.0092 | 0.0093 | | 0.0092 | | 0.0093 | | 0.0093 | 0.0092 | | 0.0093 | | 0.0093 | | 0.0092 | | 0.0092 | 0.0092 | | | 0.0093 | 0.0092 | | 0.0092 | 0.0093 | 0.0093 | 0.0093 | 0.0092 | 0.0092 | 0.0093 |
| **N_MLJNO** | | 0.0093 | | 0.0095 | | 0.0093 | | 0.0096 | 0.0096 | | 0.0095 | | 0.0090 | | 0.0091 | 0.0088 | | 0.0087 | | 0.0115 | | 0.0087 | | 0.0087 | 0.0089 | | | 0.0086 | 0.0094 | | 0.0086 | 0.0099 | 0.0109 | 0.0089 | 0.0088 | 0.0087 | 0.0090 |
| **N_MLMFD** | | 0.0092 | | 0.0095 | | 0.0093 | | 0.0092 | 0.0095 | | 0.0096 | | 0.0092 | | 0.0091 | 0.0088 | | 0.0087 | | 0.0103 | | 0.0088 | | 0.0088 | 0.0089 | | | 0.0087 | 0.0093 | | 0.0088 | 0.0096 | 0.0100 | 0.0090 | 0.0089 | 0.0088 | 0.0089 |
| **N_MLSEC** | | 0.0092 | | 0.0093 | | 0.0093 | | 0.0092 | 0.0093 | | 0.0093 | | 0.0093 | | 0.0092 | 0.0092 | | 0.0091 | | 0.0095 | | 0.0092 | | 0.0091 | 0.0091 | | | 0.0092 | 0.0093 | | 0.0092 | 0.0094 | 0.0094 | 0.0092 | 0.0092 | 0.0092 | 0.0092 |
| **N_MLSES** | | 0.0093 | | 0.0093 | | 0.0092 | | 0.0093 | 0.0093 | | 0.0093 | | 0.0093 | | 0.0092 | 0.0092 | | 0.0092 | | 0.0093 | | 0.0092 | | 0.0092 | 0.0093 | | | 0.0093 | 0.0092 | | 0.0092 | 0.0093 | 0.0093 | 0.0093 | 0.0092 | 0.0092 | 0.0092 |
| **N_MLTYR** | | 0.0093 | | 0.0093 | | 0.0092 | | 0.0093 | 0.0094 | | 0.0093 | | 0.0092 | | 0.0092 | 0.0092 | | 0.0092 | | 0.0100 | | 0.0091 | | 0.0091 | 0.0092 | | | 0.0091 | 0.0093 | | 0.0091 | 0.0094 | 0.0095 | 0.0092 | 0.0092 | 0.0091 | 0.0092 |
| **N_MLWP** | | 0.0093 | | 0.0095 | | 0.0092 | | 0.0092 | 0.0094 | | 0.0095 | | 0.0092 | | 0.0092 | 0.0090 | | 0.0089 | | 0.0101 | | 0.0090 | | 0.0090 | 0.0091 | | | 0.0090 | 0.0093 | | 0.0090 | 0.0095 | 0.0098 | 0.0092 | 0.0091 | 0.0090 | 0.0091 |
| **N_YTC** | | 0.0092 | | 0.0093 | | 0.0093 | | 0.0093 | 0.0093 | | 0.0093 | | 0.0092 | | 0.0092 | 0.0091 | | 0.0091 | | 0.0098 | | 0.0091 | | 0.0091 | 0.0091 | | | 0.0091 | 0.0093 | | 0.0091 | 0.0094 | 0.0096 | 0.0092 | 0.0091 | 0.0091 | 0.0092 |
|  |  | |  | |  | |  | | |  | |  | |  | | |  | |  | | **Migration rates into…** | | | | | |  | | |  |  |  |  |  |  |  |  |
| **I_WRN** | **I_YTS** | | **I_ZRB** | | **N_MLCP** | | **N_MLGB** | | | **N_MLHNS** | | **N_MLJNO** | | **N_MLMFD** | | | **N_MLSEC** | | **N_MLSES** | | **N_MLTYR** | | **N_MLWP** | | | **N_YTC** | **From…** | | |  |  |  |  |  |  |  |  |
| 0.0099 | 0.0089 | | 0.0091 | | 0.0092 | | 0.0090 | | | 0.0090 | | 0.0093 | | 0.0098 | | | 0.0091 | | 0.0090 | | 0.0090 | | 0.0096 | | | 0.0089 | **I_CGF** | | |  |  |  |  |  |  |  |  |
| 0.0099 | 0.0088 | | 0.0090 | | 0.0090 | | 0.0090 | | | 0.0090 | | 0.0095 | | 0.0098 | | | 0.0090 | | 0.0088 | | 0.0089 | | 0.0095 | | | 0.0089 | **I_CRN** | | |  |  |  |  |  |  |  |  |
| 0.0101 | 0.0088 | | 0.0090 | | 0.0091 | | 0.0089 | | | 0.0089 | | 0.0097 | | 0.0098 | | | 0.0089 | | 0.0088 | | 0.0089 | | 0.0096 | | | 0.0088 | **I_DAL** | | |  |  |  |  |  |  |  |  |
| 0.0099 | 0.0089 | | 0.0090 | | 0.0091 | | 0.0090 | | | 0.0090 | | 0.0095 | | 0.0097 | | | 0.0089 | | 0.0090 | | 0.0090 | | 0.0096 | | | 0.0089 | **I_FST** | | |  |  |  |  |  |  |  |  |
| 0.0098 | 0.0089 | | 0.0090 | | 0.0091 | | 0.0090 | | | 0.0090 | | 0.0095 | | 0.0098 | | | 0.0091 | | 0.0090 | | 0.0089 | | 0.0095 | | | 0.0088 | **I_KRF** | | |  |  |  |  |  |  |  |  |
| 0.0101 | 0.0088 | | 0.0091 | | 0.0091 | | 0.0089 | | | 0.0089 | | 0.0095 | | 0.0098 | | | 0.0089 | | 0.0088 | | 0.0090 | | 0.0097 | | | 0.0088 | **I_KSC** | | |  |  |  |  |  |  |  |  |
| 0.0098 | 0.0089 | | 0.0090 | | 0.0093 | | 0.0091 | | | 0.0091 | | 0.0093 | | 0.0099 | | | 0.0090 | | 0.0090 | | 0.0089 | | 0.0097 | | | 0.0089 | **I_KUI** | | |  |  |  |  |  |  |  |  |
| 0.0099 | 0.0089 | | 0.0092 | | 0.0091 | | 0.0090 | | | 0.0090 | | 0.0094 | | 0.0098 | | | 0.0090 | | 0.0090 | | 0.0090 | | 0.0096 | | | 0.0089 | **I_LUL** | | |  |  |  |  |  |  |  |  |
| 0.0096 | 0.0091 | | 0.0091 | | 0.0092 | | 0.0091 | | | 0.0091 | | 0.0095 | | 0.0094 | | | 0.0091 | | 0.0091 | | 0.0092 | | 0.0094 | | | 0.0092 | **I_MIT** | | |  |  |  |  |  |  |  |  |
| 0.0095 | 0.0091 | | 0.0092 | | 0.0092 | | 0.0091 | | | 0.0091 | | 0.0095 | | 0.0094 | | | 0.0092 | | 0.0091 | | 0.0092 | | 0.0093 | | | 0.0091 | **I_MLGB** | | |  |  |  |  |  |  |  |  |
| 0.0105 | 0.0084 | | 0.0089 | | 0.0087 | | 0.0086 | | | 0.0085 | | 0.0100 | | 0.0100 | | | 0.0085 | | 0.0086 | | 0.0089 | | 0.0097 | | | 0.0085 | **I_MLHNS** | | |  |  |  |  |  |  |  |  |
| 0.0095 | 0.0090 | | 0.0092 | | 0.0092 | | 0.0091 | | | 0.0091 | | 0.0095 | | 0.0095 | | | 0.0091 | | 0.0091 | | 0.0092 | | 0.0094 | | | 0.0091 | **I_MLJNO** | | |  |  |  |  |  |  |  |  |
| 0.0094 | 0.0092 | | 0.0092 | | 0.0092 | | 0.0092 | | | 0.0092 | | 0.0092 | | 0.0094 | | | 0.0092 | | 0.0092 | | 0.0092 | | 0.0093 | | | 0.0092 | **I_MLS** | | |  |  |  |  |  |  |  |  |
| 0.0099 | 0.0089 | | 0.0092 | | 0.0091 | | 0.0090 | | | 0.0090 | | 0.0096 | | 0.0097 | | | 0.0090 | | 0.0089 | | 0.0091 | | 0.0096 | | | 0.0091 | **I_MLSEC** | | |  |  |  |  |  |  |  |  |
| 0.0093 | 0.0092 | | 0.0092 | | 0.0093 | | 0.0092 | | | 0.0093 | | 0.0092 | | 0.0094 | | | 0.0092 | | 0.0093 | | 0.0093 | | 0.0093 | | | 0.0092 | **I_MLSEN** | | |  |  |  |  |  |  |  |  |
| 0.0098 | 0.0090 | | 0.0091 | | 0.0092 | | 0.0090 | | | 0.0091 | | 0.0094 | | 0.0096 | | | 0.0090 | | 0.0089 | | 0.0091 | | 0.0095 | | | 0.0090 | **I_NYS** | | |  |  |  |  |  |  |  |  |
| 0.0093 | 0.0092 | | 0.0093 | | 0.0093 | | 0.0092 | | | 0.0092 | | 0.0092 | | 0.0094 | | | 0.0092 | | 0.0093 | | 0.0091 | | 0.0093 | | | 0.0093 | **I_ORI** | | |  |  |  |  |  |  |  |  |
| 0.0103 | 0.0087 | | 0.0090 | | 0.0091 | | 0.0088 | | | 0.0088 | | 0.0098 | | 0.0100 | | | 0.0088 | | 0.0088 | | 0.0089 | | 0.0096 | | | 0.0087 | **I_POW** | | |  |  |  |  |  |  |  |  |
| 0.0101 | 0.0086 | | 0.0090 | | 0.0091 | | 0.0089 | | | 0.0087 | | 0.0099 | | 0.0102 | | | 0.0087 | | 0.0088 | | 0.0088 | | 0.0098 | | | 0.0087 | **I_REV** | | |  |  |  |  |  |  |  |  |
| 0.0101 | 0.0088 | | 0.0090 | | 0.0090 | | 0.0089 | | | 0.0090 | | 0.0096 | | 0.0098 | | | 0.0089 | | 0.0089 | | 0.0090 | | 0.0096 | | | 0.0089 | **I_SMZ** | | |  |  |  |  |  |  |  |  |
| 0.0093 | 0.0092 | | 0.0092 | | 0.0092 | | 0.0093 | | | 0.0093 | | 0.0093 | | 0.0093 | | | 0.0092 | | 0.0092 | | 0.0092 | | 0.0093 | | | 0.0092 | **I_SWN** | | |  |  |  |  |  |  |  |  |
| 0.0093 | 0.0092 | | 0.0093 | | 0.0093 | | 0.0092 | | | 0.0093 | | 0.0093 | | 0.0093 | | | 0.0093 | | 0.0092 | | 0.0092 | | 0.0093 | | | 0.0092 | **I_TXN** | | |  |  |  |  |  |  |  |  |
| 0.0097 | 0.0090 | | 0.0092 | | 0.0092 | | 0.0091 | | | 0.0092 | | 0.0094 | | 0.0097 | | | 0.0091 | | 0.0091 | | 0.0091 | | 0.0095 | | | 0.0091 | **I_WRG** | | |  |  |  |  |  |  |  |  |
| **0.6797** | 0.0087 | | 0.0089 | | 0.0089 | | 0.0088 | | | 0.0089 | | 0.0097 | | 0.0099 | | | 0.0087 | | 0.0087 | | 0.0088 | | 0.0096 | | | 0.0087 | **I_WRN** | | |  |  |  |  |  |  |  |  |
| 0.0096 | **0.6761** | | 0.0091 | | 0.0092 | | 0.0091 | | | 0.0091 | | 0.0094 | | 0.0095 | | | 0.0091 | | 0.0091 | | 0.0091 | | 0.0094 | | | 0.0091 | **I_YTS** | | |  |  |  |  |  |  |  |  |
| 0.0096 | 0.0090 | | **0.6762** | | 0.0091 | | 0.0091 | | | 0.0091 | | 0.0095 | | 0.0095 | | | 0.0091 | | 0.0091 | | 0.0091 | | 0.0095 | | | 0.0090 | **I_ZRB** | | |  |  |  |  |  |  |  |  |
| 0.0096 | 0.0091 | | 0.0091 | | **0.6771** | | 0.0092 | | | 0.0091 | | 0.0093 | | 0.0098 | | | 0.0091 | | 0.0090 | | 0.0090 | | 0.0096 | | | 0.0090 | **N_MLCP** | | |  |  |  |  |  |  |  |  |
| 0.0093 | 0.0092 | | 0.0092 | | 0.0093 | | **0.6765** | | | 0.0093 | | 0.0093 | | 0.0094 | | | 0.0093 | | 0.0092 | | 0.0092 | | 0.0093 | | | 0.0092 | **N_MLGB** | | |  |  |  |  |  |  |  |  |
| 0.0093 | 0.0092 | | 0.0093 | | 0.0093 | | 0.0092 | | | **0.6762** | | 0.0092 | | 0.0093 | | | 0.0092 | | 0.0092 | | 0.0092 | | 0.0093 | | | 0.0092 | **N_MLHNS** | | |  |  |  |  |  |  |  |  |
| 0.0104 | 0.0086 | | 0.0090 | | 0.0090 | | 0.0087 | | | 0.0085 | | **0.6781** | | 0.0102 | | | 0.0086 | | 0.0087 | | 0.0089 | | 0.0098 | | | 0.0087 | **N_MLJNO** | | |  |  |  |  |  |  |  |  |
| 0.0101 | 0.0087 | | 0.0090 | | 0.0091 | | 0.0090 | | | 0.0089 | | 0.0095 | | **0.6797** | | | 0.0089 | | 0.0088 | | 0.0088 | | 0.0099 | | | 0.0087 | **N_MLMFD** | | |  |  |  |  |  |  |  |  |
| 0.0094 | 0.0092 | | 0.0092 | | 0.0093 | | 0.0092 | | | 0.0092 | | 0.0092 | | 0.0095 | | | **0.6764** | | 0.0092 | | 0.0092 | | 0.0093 | | | 0.0092 | **N_MLSEC** | | |  |  |  |  |  |  |  |  |
| 0.0093 | 0.0092 | | 0.0092 | | 0.0093 | | 0.0092 | | | 0.0092 | | 0.0093 | | 0.0093 | | | 0.0092 | | **0.6762** | | 0.0092 | | 0.0093 | | | 0.0092 | **N_MLSES** | | |  |  |  |  |  |  |  |  |
| 0.0096 | 0.0091 | | 0.0092 | | 0.0092 | | 0.0091 | | | 0.0092 | | 0.0094 | | 0.0094 | | | 0.0091 | | 0.0091 | | **0.6761** | | 0.0093 | | | 0.0091 | **N_MLTYR** | | |  |  |  |  |  |  |  |  |
| 0.0099 | 0.0089 | | 0.0091 | | 0.0092 | | 0.0090 | | | 0.0090 | | 0.0093 | | 0.0099 | | | 0.0090 | | 0.0090 | | 0.0090 | | **0.6772** | | | 0.0089 | **N_MLWP** | | |  |  |  |  |  |  |  |  |
| 0.0095 | 0.0091 | | 0.0092 | | 0.0092 | | 0.0092 | | | 0.0091 | | 0.0094 | | 0.0095 | | | 0.0092 | | 0.0091 | | 0.0092 | | 0.0094 | | | **0.6761** | **N_YTC** | | |  |  |  |  |  |  |  |  |

Supplemental Table 7. Cyt *b* and phased multilocus divergence date estimates.

|  | **cyt*b*** | | |  | **multilocus** | | |
| --- | --- | --- | --- | --- | --- | --- | --- |
| **Lineage** | **95% HPD lower** | **mean** | **95% HPD upper** |  | **95% HPD lower** | **mean** | **95% HPD upper** |
| *M.longicaudus* | 775,600 | 1,115,100 | 1,561,100 |  | 184,200 | 489,800 | 1,890,900 |
| Southern | 371,700 | 727,600 | 1,106,900 |  |  |  |  |
| S1 | 160,800 | 307,900 | 487,200 |  |  |  |  |
| S2 | 23,737 | 78,589 | 142,200 |  |  |  |  |
| Central/North/Island | 459,600 | 661,200 | 906,300 |  |  |  |  |
| Central | 128,500 | 217,000 | 314,400 |  |  |  |  |
| COP | 171,400 | 294,300 | 420,500 |  |  |  |  |
| North/Island | 306,400 | 435,400 | 581,900 |  | 101,700 | 142,400 | 213,100 |
| NPC | 169,500 | 294,200 | 169,500 |  |  |  |  |
| North | 105,000 | 159,000 | 221,600 |  |  |  |  |
| Island | 164,600 | 225,100 | 294,200 |  |  |  |  |

Supplemental text:

**Methods**

*Phylogenetic analyses and estimation of divergence times*

An uncorrelated lognormal relaxed clock was employed for cyt *b* at a mutation rate of 4% Myr^-1^ and strict clocks were set for estimations of phased nuclear loci based on cyt *b*. Utilizing a Bayesian uncorrelated relaxed clock assists in reducing errors associated with recent divergence and the lack of reliable calibration points (Drummond *et al.* 2006; Ho & Duchene 2014).

Phylogenetic reconstructions using cyt *b* were estimated within Maximum Likelihood (ML) and Bayesian frameworks. The TrN+I+G model of evolution had the lowest AIC value using ModelTest (Posada & Buckley 2004; Posada & Crandall 1998). ML estimations were performed in mega v5.2 (Tamura *et al.* 2011) with 1,000 bootstrap replicates. To generate the Bayesian phylogeny and divergence dates for major clades we used Beast v1.7.5 (Drummond *et al.* 2012) and input files prepared in BEAUti v1.7.5. We applied a coalescent constant-size (Kingman 1982) tree prior with a random start tree, using an uncorrelated lognormal relaxed clock for 60 million generations (sampled every 2000). Tracer, LogCombiner and TreeAnnotator were used as in main text.

Net genetic divergence among major cyt *b* clades was calculated in mega v5.2 (Tamura *et al.* 2011).

**Results**

*Phylogenetic analysis*

Mean net genetic distance (Supplemental Table 3) within *M. longicaudus* cyt *b* was 2.7% ± 0.4%, with least divergence between Northern and NPC (0.5% ± 0.1%) and largest divergence between S1 and Northern, NPC, Island, and Central clades (3.3% ± 0.4%). Consistent with genetic distances, gene flow values between 0.035 and 0.10 are within typically observed values for inter- and intra- specific estimates (e.g., Nakajima *et al.* 2012; Ross *et al.* 2010).

*Divergence times and alternate models of glacial refugia and postglacial recolonization*

Using an average rodent mutation rate, cyt *b* TMRCAs detected for *M. longicaudus* and all clades (except S2) were before the LIG, and for S2 before the LGM (Supplemental Table 6).
